# Genetic Analysis Using a Multi-Parent Wheat Population Identifies Novel Sources of Septoria Tritici Blotch Resistance

**DOI:** 10.3390/genes11080887

**Published:** 2020-08-04

**Authors:** Adnan Riaz, Petra KockAppelgren, James Gerard Hehir, Jie Kang, Fergus Meade, James Cockram, Dan Milbourne, John Spink, Ewen Mullins, Stephen Byrne

**Affiliations:** 1 Teagasc, Crop Science Department, Oak Park, R93 XE12 Carlow, Ireland; adnan.riaz@teagasc.ie (A.R.); petra@farmingrathcroghan.ie (P.K.); ghehir@gmail.com (J.G.H.); Jie.Kang@agresearch.co.nz (J.K.); Fergus.Meade@teagasc.ie (F.M.); Dan.Milbourne@teagasc.ie (D.M.); John.Spink@teagasc.ie (J.S.); Ewen.Mullins@teagasc.ie (E.M.); 2 AgResearch, Invermay Agricultural Centre, Private Bag, Mosgiel 50034, New Zealand; 3 Department of Mathematics and Statistics, University of Otago, Dunedin 9016, New Zealand; 4 The John Bingham Laboratory, NIAB, Cambridge CB3 0LE, UK; James.Cockram@niab.com

**Keywords:** wheat, MAGIC population, septoria tritici blotch, genetic disease resistance, quantitative trait locus (QTL) mapping, SNP genotyping array, r/qtl2

## Abstract

*Zymoseptoria tritici* is the causative fungal pathogen of septoria tritici blotch (STB) disease of wheat (*Triticum aestivum* L.) that continuously threatens wheat crops in Ireland and throughout Europe. Under favorable conditions, STB can cause up to 50% yield losses if left untreated. STB is commonly controlled with fungicides; however, a combination of *Z. tritici* populations developing fungicide resistance and increased restrictions on fungicide use in the EU has led to farmers relying on fewer active substances. Consequently, this serves to drive the emergence of *Z. tritici* resistance against the remaining chemistries. In response, the use of resistant wheat varieties provides a more sustainable disease management strategy. However, the number of varieties offering an adequate level of resistance against STB is limited. Therefore, new sources of resistance or improved stacking of existing resistance loci are needed to develop varieties with superior agronomic performance. Here, we identified quantitative trait loci (QTL) for STB resistance in the eight-founder “NIAB Elite MAGIC” winter wheat population. The population was screened for STB response in the field under natural infection for three seasons from 2016 to 2018. Twenty-five QTL associated with STB resistance were identified in total. QTL either co-located with previously reported QTL or represent new loci underpinning STB resistance. The genomic regions identified and the linked genetic markers serve as useful resources for STB resistance breeding, supporting rapid selection of favorable alleles for the breeding of new wheat cultivars with improved STB resistance.

## 1. Introduction

Bread or common wheat (*Triticum aestivum* L.) is a primary tillage crop in Ireland and the most important cereal crop in the European Union (EU), accounting for 46% of EU-28 cereal production [1]. Annual wheat yield losses are observed due to extreme weather patterns such as heat, heavy rainfall, drought, and nutrient deficiency [2,3]. For instance, in 2018, a widespread drought was observed in central and northern Europe; consequently, wheat production dropped by 9.5%, along with a slight decline in area under cultivation of 1.6% [1]. Furthermore, frequent emergence and re-emergence of pests and diseases are a constant threat to wheat yields [4,5,6].

**Figure 1:**
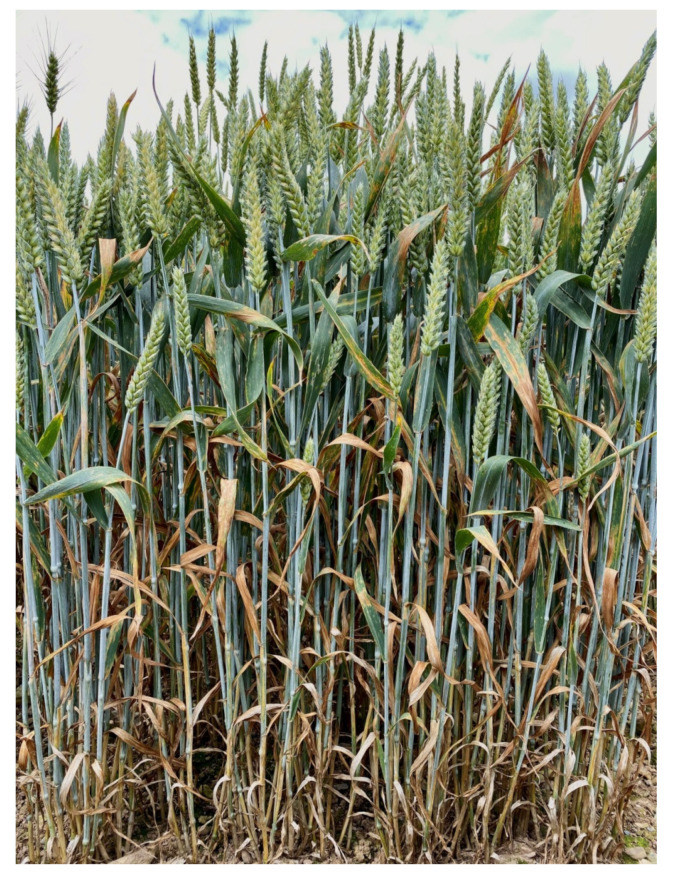
Septoria tritici blotch (STB) disease of wheat in a field trial in Carlow, Ireland, showing the resulting necrosis of the leaves within the crop canopy.

Septoria tritici blotch (STB), caused by the necrotrophic fungus *Zymoseptoria tritici*, is one of the most devastating foliar diseases of wheat in Ireland and Europe [7]. STB epidemics can cause a yield loss of up to 50% in fields when a susceptible wheat cultivar is grown [8,9]. Yield losses vary with environmental conditions, variety, and cropping system. STB severity on flag and flag-1 leaves result in a reduction of the green leaf area, limiting plant growth and reducing grain size, which ultimately impacts on yield and grain quality [10,11]. *Z. tritici* produces ascospores and pycnidiospores via a sexual [12] and an asexual life cycle [13], respectively. Both types of spores can serve as primary inoculum depending upon environmental conditions, cropping history, and management [14]. Ascospores survive on crop stubble and are dispersed by wind, thus commonly serving as the primary inoculum [14]. The polycyclic pycnidiospores are dispersed by rain-splash, thus helping to propagate STB epidemics through the wheat growing season [15,16]. The progress of an STB epidemic depends on the availability of optimal temperature (22 °C) [17] and relative humidity (≥85%) [18], which promotes the appearance of black asexual fruiting bodies (pycnidia) that in turn disperse pycnidiospores from infected leaves [19]. STB progression is characterized by an asymptomatic latent phase that may vary between 14–28 days under field conditions and 9–14 days under controlled environmental conditions, depending on host, isolate, and prevailing environmental conditions [20,21,22,23,24,25,26,27]. After 10–12 days post-infection, the pathogen transitions to a necrotrophic phase, which is usually characterized by brown coloured lesions on wheat leaves that typically develop into necrotic irregularly-shaped blotches (Figure 1).

At present, the two main methods to control STB are the use of fungicides and resistant cultivars. However, there is an over-dependence on fungicide application, with control of STB accounting for more than 70% of annual fungicide usage in Europe, the use of which costs the sector over €1 bn per annum [8]. Over the years, worldwide *Z. tritici* populations have developed fungicide resistance [28,29,30]. Additionally, within the EU, fungicide registrations are being reversed (EU Reg. No 1107/2009). The result is that fewer active substances are available to farmers, leading to challenges in efficient wheat production. The use of resistant wheat varieties provides a more economically and environmentally sustainable disease management strategy. In total, 22 resistance genes (*Stb*; *Stb1-Stb19*, *StbSm3*, *StbWW*, and *TmStb1*) and 89 quantitative trait loci (QTL) have been identified for STB resistance to date, mainly in wheat (for detailed review [31]). There are two types of STB resistance genes, namely qualitative and quantitative. For instance, *Stb6*, one of the two most commonly deployed resistance genes in European germplasm [32], encodes a wall-associated receptor kinase (WAK)-like protein [33] conferring resistance against *Z. tritici* isolate “IPO323”, which carry the corresponding avirulence gene *AvrStb6* [34]. However, *Stb6* alone is not able to confer resistance under field conditions [35]. In contrast, *Stb16q* confers quantitative resistance at both seedling and the adult plant stage against multiple *Z. tritici* isolates [36]. *Z*. *tritici* can evolve rapidly, adapting to chemical control measures and deployed wheat resistance genes due to its host adaptable genome [37,38], sexual recombination [39] and gene flow [40]. For instance, virulence to *Stb16q* has recently been detected in Irish [41] and French [42] *Z. tritici* populations. Although a large number of known STB resistance genes and QTL are available, there is a continuous drive to discover and characterise new sources of resistance against *Z. tritici* for use in elite wheat germplasm.

Genetic resistance to STB is underpinned by complex genetic control, thus making it challenging to dissect the underlying genetic architecture. Therefore, specialized populations using two founders that are often selected due to contrasting STB resistance are typically developed to identify and genetically map genes conferring resistance. However, traditional bi-parental populations have limited allelic diversity at a given locus (i.e., as just two parents contribute variants), and map resolution is limited by a potentially lower level of genetic recombination, typically via a single round of inter-crossing [43]. Alternatively, multi-parent populations such as nested association mapping (NAM) [44] and multi-parent advanced generation inter-cross (MAGIC) [45] overcome some of these limitations. A MAGIC population typically comprises 4, 8, or 16 founder parents selected for various desirable traits, such as disease resistance, plant height, flowering time, and yield. The founders are subjected to several generations of intercrossing (e.g., eight-founder populations require three rounds of inter-crossing to combine genomic contributions from all founders), followed by multiple generations of selfing to create recombinant inbred lines (RILs) that each carry a mosaic of the founder haplotypes [46]. MAGIC populations have been developed for numerous plant species, including arabidopsis [47], rice [48], wheat [45,49], barley [50], maize [51], and tomato [52]. Multi-parent populations can provide an improved understanding of the genetic architecture of complex traits due to increased recombination, segregation of multiple alleles, relatively high mapping resolution, and the possibility of allowing pleiotropic QTL and closely linked QTL to be distinguished [53].

The multiple rounds of founder intercrossing and RIL inbreeding combined with relatively large population sizes mean that the use of MAGIC populations have the potential to provide high genetic mapping resolution and therefore can be used for both fine and coarse mapping, particularly in inbreeding species such as rice and wheat [54]. The first wheat MAGIC population was developed from four elite Australian cultivars and generated a population of 1579 lines. Genomic regions controlling quantitative traits such as plant height and hectoliter weight were identified in the population [45]. Since then, additional wheat MAGIC populations have been generated, including the “NIAB Elite MAGIC population” [49], the “NCCR durum wheat population” [55], the “MAGIC winter wheat population” (WM-800) [56], and the “Bavarian MAGIC” wheat population (BMWpop) [57]. These MAGIC populations have been exploited to identify different genomic regions underpinning yield and other agronomic traits. For instance, the eight founder “NIAB Elite MAGIC” winter wheat population was screened in diverse environments for 18 different yield and agronomic traits [49]. A total of 376 QTL were identified, of which 20 were developed into Kompetitive allele-specific primer (KASP) genetic markers for potential use in marker-assisted selection in wheat breeding programmes [58].

Different wheat MAGIC populations have been exploited to identify QTL controlling resistance against fungal diseases such as powdery mildew (caused by *Blumeria graminis*), STB, tan spot (caused by *Pyrenophora tritici-repentis*) [59,60], and Septoria leaf and glume blotch (caused by *Parastagonospora nodorum*) [61]. While genetic resistance to STB has been previously identified using BMWpop [60], the phenotypic response was observed under artificial inoculation. Given that the *Z. tritici* pathogen population is rapidly evolving, it is important to screen the genetic resources such as MAGIC populations under natural STB infection in field conditions.

In this study, the “NIAB Elite MAGIC” eight-founder winter wheat population [49] was investigated for STB response under natural infection in the field across three years in Ireland. We identified genomic regions through QTL mapping underpinning STB resistance in the MAGIC population.

## 2. Materials and Methods

### 2.1. Plant Material

The structure and the design of the “NIAB Elite MAGIC” population has been previously described [49]. Briefly, it was developed from eight wheat cultivars, namely Alchemy, Brompton, Claire, Hereward, Rialto, Robigus, Soissons, and Xi19 [49]. The founders were intercrossed for three generations, followed by multiple rounds of selfing to produce RILs.

### 2.2. Field Trials

The MAGIC population was evaluated for three field seasons from 2015–2016, 2016–2017, and 2017–2018 in the field trial area at Teagasc (52°51′21.4″ N 6°54′51.3″ W), Oak Park, Carlow, Ireland (Table 1). Trials were sown in the Autumn and reached maturity the following summer. Subsequently, we refer to 2015–2106, 2016–2017, and 2017–2018 trials as the 2016, the 2017, and the 2018 trials, respectively. All field trials were managed using a standard agronomy program for the trial site location, including the use of fertilizers and chemical fungicides (Table 1). The STB susceptible variety “JB Diego” was planted at trial borders to help pathogen spread and maximise the epidemic pressure.

#### 2.2.1. 2016 Field Trial

In 2016, a total of 1076 lines of the “NIAB Elite MAGIC” population were assessed for STB response, including the eight founders and four reference winter wheat varieties (Table S1). An α lattice design was implemented in which each line was present in each of three blocks. Within each block, 34 sub-blocks contained 32 plots (four plots wide by eight plots long); each plot was a single row of 0.95 m length × 0.2 m width. The population was screened under natural STB infection from the end of June to mid-July at two-time points (i.e., T1 and T2) separated by fourteen days. STB severity was assessed as the percentage of leaf area covered by lesions on the top three leaves (i.e., flag, flag-1, and flag-2) on the first time point, while only flag leaves were evaluated at the second time point. Plant height (cm) and flowering time (i.e., days from sowing to flowering) data were also collected. Plant height (from stem base to the tip of ear) was measured on four randomly selected tillers per plot between Zadocks growth stages GS73 (early milk) to GS85 (soft dough). Average plant height was calculated and used in the statistical analysis. Flowering time was scored when 50% of the plants in a plot reached GS69 (anthesis complete) [62] (Table 1).

#### 2.2.2. 2017 Field Trial

In 2017, a subset of the MAGIC population (192 lines) was sown in a replicated trial with larger plots together with the founder lines and the disease standards (Table S1). Lines were selected from almost all of the 208 eight-way families in the MAGIC population (a single line from each of 192 of the eight-way families was selected). An α lattice design was used, and lines were evaluated in two blocks (two replications) where each block was further divided into 40 rows and 10 columns. Each line was sown in plots 2.5 m length × 1.2 m wide, consisting of six rows per plot. The gap between the adjacent plots was 0.4 m. STB assessment was carried out twice in the season after GS69 with a difference of 14 days between assessments. STB severity (i.e., percentage of disease area of the leaf) measurements were taken on the top three leaves (i.e., flag, flag-1, and flag-2) using four randomly selected tillers per plot. Plant height and flowering time were also recorded for the whole experiment, as described above (Table 1).

#### 2.2.3. 2018 Field Trial

In 2018, 388 MAGIC lines were grown, as well as the eight founder lines and twelve commercially available cultivars (Table S1). As in 2017, the 388 lines were selected to represent almost all of the 208 eight-way families in the MAGIC population. The trial comprised three blocks, where each line plot contained a single row 0.95 m length × 0.2 m wide. The plants were assessed once for STB severity on the top three leaves (i.e., flag, flag-1, and flag-2) using three randomly selected tillers per plot. Plant height and flowering time data were not recorded in 2018. Therefore, plant height and flowering time data from 2016 were used in the statistical analysis; these traits are highly heritable [63].

### 2.3. Phenotypic Data Analysis

The phenotypic data were analysed in R [64]. Each data collection time point in a year was treated as an individual dataset and analysed separately. Phenotypic data were transformed to a normal distribution using the logit (p) function (Figure S1). In 2016, plant height and flowering time were recorded for a single replication. The phenotypic correlations were calculated to understand the concordance of STB disease scores measured on different leaves at different time points and in different years. We also calculated the correlation between STB datasets and plant height and flowering time to understand the relationship between STB disease scores and these developmental traits. The correlations were calculated using Pearson correlation coefficients in the Hmisc package [65]. The logit-transformed phenotypic values were analysed in a linear mixed model using R/lme4 [66,67].

Within each dataset, the phenotypic data were adjusted based on the following model:

$$y_{ijk}=\mu +g_{i}+b_{j}+s_{kj}+\epsilon_{ijk}$$

where $y_{ijk}$ is the observed STB score of each line, μ is the overall mean, $g_{i}$ is the fixed effect of line *i*, $b_{j}$ describes the random effect of block *j*, $s_{kj}$ is the random effect of subblock *k* nested within the block *j*, and $\epsilon_{ijk}$ is the error term. The adjusted STB means were estimated through the model and were used in subsequent QTL analysis. In the case of the founder lines, each STB dataset was logit (p) back-transformed to a 0–100 percentage scale for presentation.

### 2.4. QTL Mapping

A subset of 643 RILs from the “NIAB Elite MAGIC” population was previously genotyped at the F_5_ generation using a 90K single nucleotide polymorphism (SNP) array, resulting in 20,643 polymorphic SNPs [49,68]. A genetic map comprising 18,601 SNPs was subsequently developed [68]. Quality control was implemented, such as removing monomorphic markers, markers with minor allele frequency (MAF) ≤ 0.05, and markers with more than 5% of missing data were removed. Lines having more than 50% missing data were removed from the analysis. A subset of 4988 markers having unique map positions, and which were common between the MAGIC population genotypes used in this study and the associated genetic map, was used for QTL mapping using the R/qtl2 package [69]. Phenotypic data, genotypic data, pedigree information, covariate data (i.e., a numeric matrix of interactive covariates—plant height and flowering time data), and genetic map were read in R/qtl2 using read_cross2(). The MAGIC population used in this study has been developed to produce a population with uniform kinship relationships [49]. In this study, pedigree information is provided for each individual in the population, consisting of a matrix of integers with individual rows and with the number of columns depending on the cross type (i.e., an eight-way magic population was defined as “riself8”). Using the information provided above, genotype probabilities were calculated with the function calc_genoprob() using multipoint SNP through the hidden Markov model (HMM). In case of eight-way MAGIC, the HMM provides a probability for each possible 36-state genotype probabilities at each marker for each line. The genotype probability information is used in estimating the kinship matrix, calculated using function calc_kinship(), which was later used to account for population structure. In case of genome scan through linear mixed model using “LOCO—leave one chromosome out” method, an argument type = “loco” was added in calc_kinship() function to calculate kinship matrix. QTL analysis using these founder haplotype probabilities was carried out via function scan1(). In the scan1() function, an argument “intcovar” was provided to account for epistatic interactions of plant height and flowering time for each dataset. Genome scans were performed using three methods: (a) by Haley-Knott regression, (b) by a linear mixed model using standard kinship matrix, and (c) by a linear mixed model using LOCO kinship matrix. To establish the statistical significance of a QTL at a 5% level of significance (α = 0.05), we performed a permutation test using the function scan1perm() where the number of permutations was 1000 and an argument “intcovar” was provided. A second threshold was set to detect weak QTL at an arbitrary threshold (the logarithm of odds (LOD) = 10), where a clear differentiation of LOD score was visible. The estimated effects of permutation significant QTL were calculated using scan1blup(), where QTL were treated as random effects and used the kinship matrix estimating residual polygenic effect. QTL were named according to standard nomenclature [70].

### 2.5. Gene Annotation of Significant Markers

Physical map positions of selected SNPs on the wheat reference genome assembly, IWGSC RefSeq v1.0 [71], were identified using The Triticeae Toolbox (T3) database [72]. The genes underlying peak SNPs were obtained from the RefSeq v1.0 assembly annotated with the RefSeq v1.0 and v1.1 gene models using JBrowse [73].

### 2.6. Alignment of QTL Identified in This Study with Previously Reported Stb Genes and QTL

The QTL identified were compared with previously reported *Stb* genes and QTL by projecting onto the physical map in The Triticeae Toolbox (T3) database to identify the co-located genes/QTL and determine the novelty of others. For our QTL co-location study, only QTL mapping and genome-wide association scan (GWAS) studies of STB using high-throughput marker platforms were considered [60,74,75,76,77,78,79,80,81,82,83]

## 3. Results

### 3.1. Field STB Response

The STB severity data from all datasets were skewed (i.e., right or left) and were therefore logit (p) transformed to improve the normality and the homoscedasticity of data (Figure S1). A significant negative correlation was observed between STB severity and plant height and flowering time for the different datasets (Table 2). The negative correlation between mean STB severity and plant height within years ranged between r = −0.2 to r = −0.29 (*p* = 0). Similarly, a significant negative correlation was identified between STB severity and flowering time for different datasets (ranging between r = −0.09 to r = −0.29 (*p* = 0; Table 2). The distribution of adjusted STB scores is shown in Figure S2. A significant positive correlation was predominantly observed between STB responses in different years (Table 3).

In 2016, a total of 1072 lines were evaluated on different leaf layers and at different time points (2016_T1_flag, 2016_T1_flag-1, 2016_T1_flag-2, and 2016_T2_flag), and the population mean for STB infection ranged between 0.11 to 86.34% (Table 4). Significant positive correlations were observed between leaves at the first time point (T1), ranging between r = 0.49 to r = 0.671 (*p* = 0; Table 3). However, the correlation between T1 and T2 flag leaf STB scores in 2016 (datasets 2016_T1_flag and 2016_T2_flag) was r = 0.3 (*p* = 0; Table 3). In 2017, a total of 191 lines were analysed. The population mean at the first and the second time points ranged from 0.35 to 65.69%, and 3.42 to 92.90%, respectively (Table 4). The correlation between the first and the second time points was r = 0.55 (flag), r = 0.77 (flag-1), and r = 0.51 (flag-2) (*p* = 0; Table 3). In 2018, a total of 374 lines were screened in all three leaf datasets (i.e., 2018_T1_flag, 2018_T1_flag-1, and 2018_T1_flag-2), and the population mean ranged between 0.26 and 87.28% (Table 4). A significant positive correlation was observed between different datasets ranging from r = 0.62 to r = 0.83 (*p* = 0; Table 3). The phenotypic data from all datasets are provided in Table S1. The broad sense heritability ranged between h^2^ = 0.14–0.217 (2016), 0.16–0.51 (2017) and 0.27–0.36 (2018).

Among the eight MAGIC founders, variable STB responses were observed in different datasets (Table 4). Soissons showed the highest mean STB severity (38.52%), while Xi19 showed the lowest (dataset 2016_T2_flag, 12.19%; Table 4). In 2017, in dataset 2017_T1_flag-2, all founders had low mean STB severity except Rialto and Soissons (Table 4). At the second time point, higher mean STB severity at 2017_T2_flag-2 was observed for all founders. Soissons showed the highest mean STB severity in the 2017_T2_flag-1 and the 2017_T2_flag-2 datasets (Table 4). The founder’s Robigus displayed low mean disease severity in dataset 2017_T2_flag leaf at the second time point (Table 4). Xi19 and Soissons were not grown in 2018 (Table 4). In the 2018_T1_flag and the 2018_T1_flag-1 disease assessments, there were limited visible symptoms of STB; however, for dataset 2018_T1_flag-2, there were higher visible STB symptoms across the founders and the population. Rialto displayed more symptoms of STB infection, while Claire displayed the lowest mean STB severity in all datasets in 2018 (Table 4). Soissons consistently displayed higher mean STB severity in all the datasets in three years (Table 4).

### 3.2. QTL Mapping

**Figure 2:**
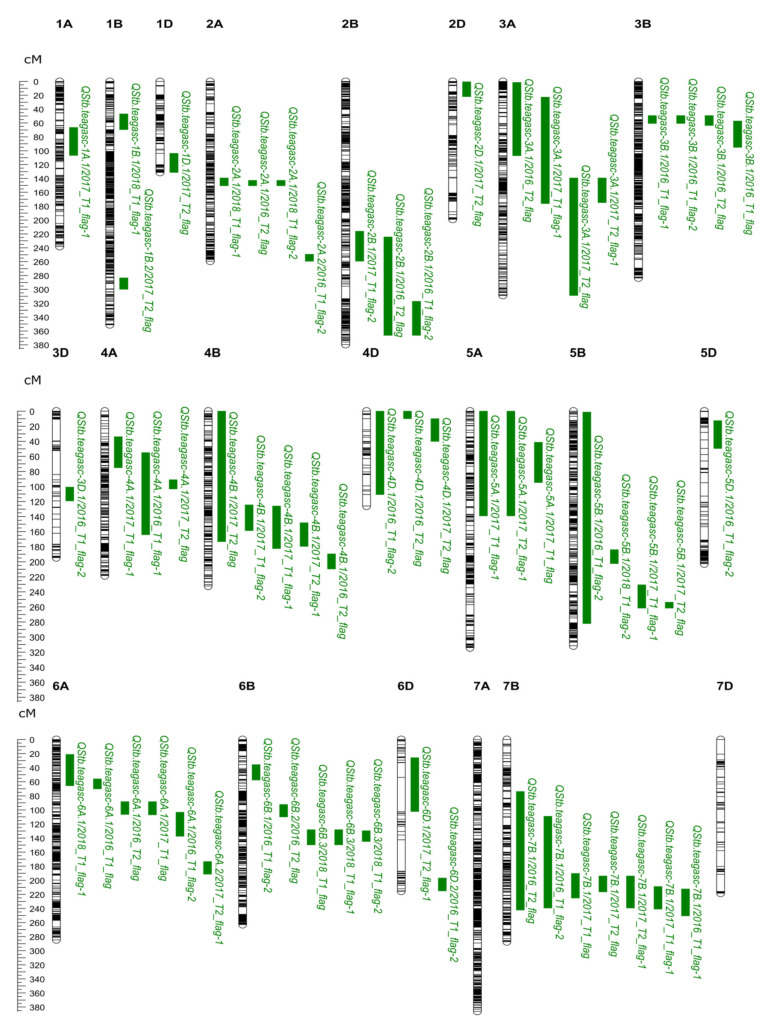
Genetic map locations (centiMorgan - cM) of septoria tritici blotch (STB) QTL detected in the “NIAB Elite MAGIC” population. QTL locations and interval sizes are indicated by the green bars on the right-hand side of each chromosome and are based on the genetic marker information in Table 5. The genetic map locations are based on the “NIAB Elite MAGIC” genetic map [68]. The QTL name assigned in this study (presented in Table 5) is followed by the environment in which the QTL was detected.

After quality control, lines with both phenotypic and genotypic data were used for QTL analysis (2016: 592 lines, 2017: 168 lines, and 2018: 333 lines). The QTL identified in different datasets but located at overlapping chromosomal positions were grouped and assigned the same name using the nomenclature *QStb.teagasc-* followed by the chromosome designation (Table 5). A total of 25 QTL associated with STB resistance were identified across the different datasets (Table 5; Figure 2). Of these, four QTL detected in four datasets were significant at the threshold determined via permutation (Table 5), while the remaining 21 QTL were identified using an arbitrary threshold (LOD = 10.0) (Table 5). Three QTL mapping approaches were used, where all QTL detected with a Haley-Knott regression model were also identified with the LMM and the LOCO models (Figure S3). The significant LOD threshold (determined via a permutation test) for each dataset at a 5% genome-wide significance level ranged between a LOD of 12.39 and a LOD of 15.40 (Table 5).

Overall, the majority of QTL were small effects accounting for 7.49–29.52% of the phenotypic variance. No QTL were consistently identified in all 13 datasets. However, a QTL on chromosome 7B, termed here *QStb.teagasc-7B.1*, was detected in seven datasets and explained 8.63–29.52% of the phenotypic variation. SNP marker BobWhite_c2892_167 at *QStb.teagasc-7B.1* had the highest LOD score (LOD = 13.8) in the 2016_T1_flag-1. Overall, a total of 25 QTL for STB were detected across the different datasets (Table 5; Figure 2). The predicted allelic effects for all permutation significant QTL are shown in (Figure S4).

The results of the QTL analysis of plant height and flowering time data for the three trials (2016, 2017, and 2018) are presented in Table S3. The QTL analysis identified significant QTL for plant height on chromosome 4B and 4D in 2016 and 2018. The plant height QTL on 4D was also detected in 2017; however, it was only identified as significant at the arbitrary threshold. Similarly, significant QTL for flowering time were detected on chromosomes 2D, 3A, 4A, and 7B in 2016, while only the chromosome 2D QTL was detected in 2017 and 2018. For comparative analysis, the flowering time and the plant height QTL identified in this study were also detected in QTL analysis for the MAGIC population using corresponding data from Scutari et al. [84] (Table S3).

### 3.3. In-Silico Gene Annotation Data and Alignment of QTL and Genes

The peak SNPs of a small number of QTL were coincidentally located with genes annotated for molecular function (Table S4). These genes were functionally annotated to identify their potential function in different process including function in modifying wall lignin-1/2, RNA 5’ end processing, cellular metabolic process, ion transmembrane transport, and defense response (Table S4).

**Figure 3:**
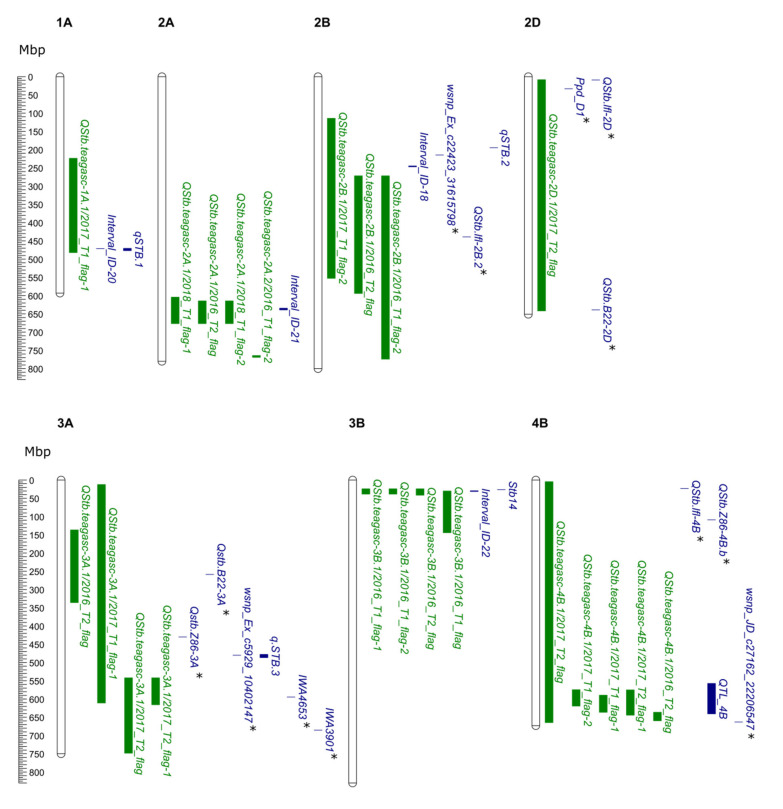

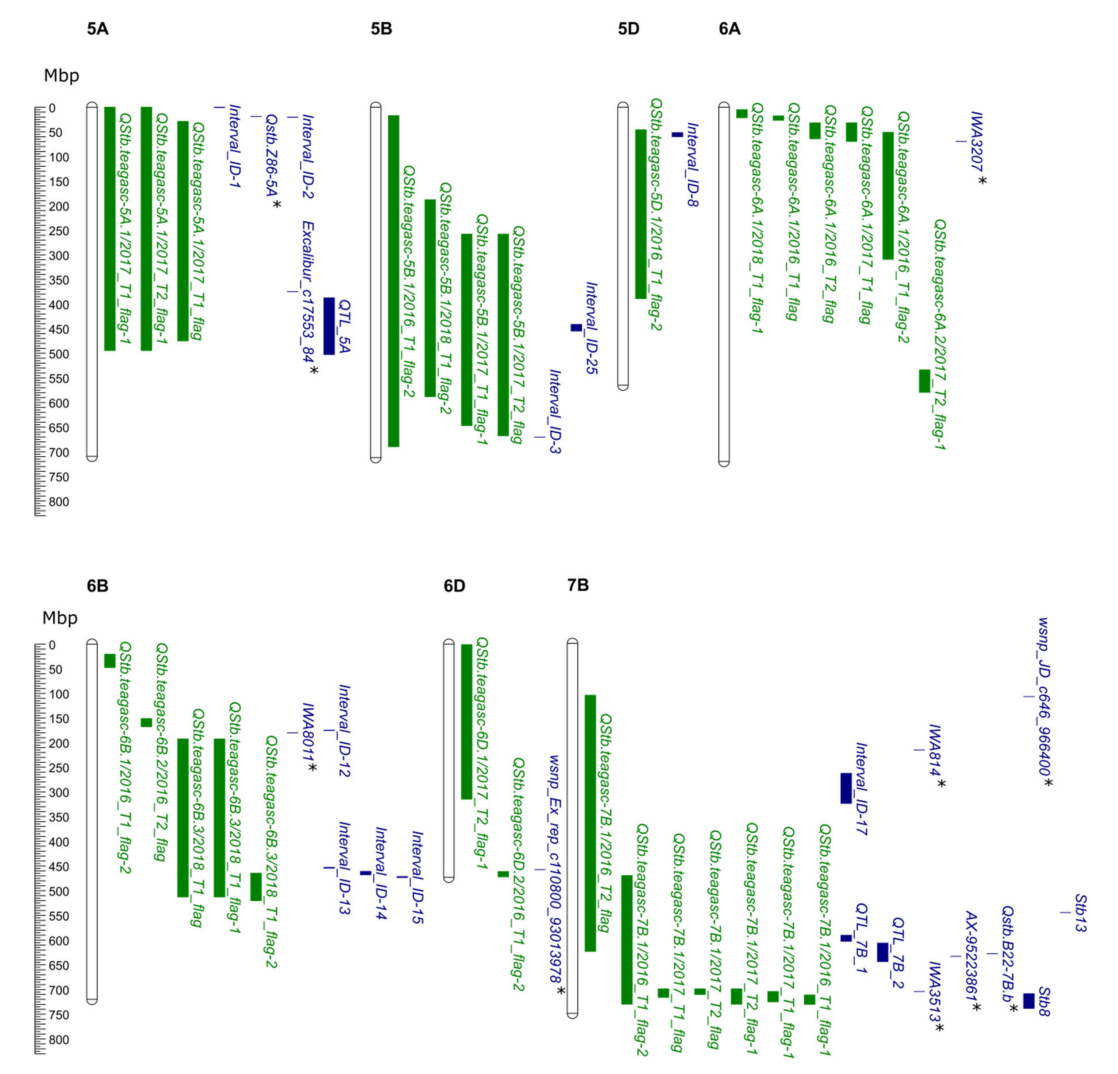
Physical map locations (megabase pair—Mbp) of septoria tritici blotch (STB) QTL detected in the current study compared to previously reported QTL. QTL locations and interval sizes are indicated by bars on the right-hand side of each chromosome and are based on the physical map information in Table 5 and Table S5. The physical map location was based on the wheat cv. Chinese Spring IWGSC RefSeq v1.0 genome assembly (IWGSC, 2018). The QTL name assigned in this study (presented in Table 5; green colour) is followed by the environment in which the QTL was detected. Previously reported QTL are indicated in blue, where the displayed QTL are only defined on the physical map by anchoring one of the two flanking makers indicated by a “*”.

Out of the 25 QTL identified, we found 15 to be co-located with previously reported QTL and *Stb* genes (i.e., *Stb8* [85], *Stb13* [86], and *Stb14* [86]), based on the anchoring of genetic markers to the wheat reference genome assembly. The remaining ten QTL were identified as potential novel loci conferring STB resistance in the field (Table S5; Figure 3).

## 4. Discussion

We screened the “NIAB Elite MAGIC” population for STB resistance under natural STB infection in field conditions for multiple years to further elucidate the complex genetic architecture underlying resistance. STB is a major wheat disease around the world, and farmers are heavily reliant on the use of fungicide programmes to control yield losses [87,88]. The increased reliance on chemical-control measures has led to the development of fungicide resistance in *Z. tritici* populations, increasing the risk for farmers and making the crop less sustainable in the long term. Alternatively, the deployment of cultivars with increased genetic resistance would provide an economic and environmentally sustainable strategy as part of an integrated management strategy. In support of this, we evaluated the “NIAB Elite MAGIC” population in an environment with typically high STB disease pressure and identified thirteen QTL associated with STB resistance.

### 4.1. Field Trials and Natural Septoria Infection

In this study, the number of lines tested varied across the years. In the first year of evaluation, the whole population of 1072 lines was planted in single-row plots. In the second year of evaluation, a subset of 191 lines was selected by taking a single line from 191 of the 208 eight-way families; within eight-way families, lines were selected based on their STB response in 2016. In the second year, lines were planted in six-row plots. In the third year, we went back to single-row plots and evaluated 374 lines; again, these were selected to represent all eight-way families. Taking a subset of the MAGIC population to represent all eight-way families was successfully used in a recent study to identify QTL for leaf and glume blotch [61]. *Z. tritici* can evolve rapidly through a high degree of recombination within and among populations, resulting in high variability in population composition [15,89], leading to variable STB responses across and within seasons.

Testing the MAGIC population under natural infection across multiple years enabled us to identify potentially durable sources of adult-plant resistance. Our evaluation site in Carlow, Ireland routinely has a medium-to-high STB inoculum pressure [88], and multi-year testing allowed us to determine stable resistances against STB populations that may differ over the years trialed. The success of an STB epidemic depends on favourable environmental conditions [8]. We see this in our study where higher STB severity was observed in 2016 and 2017 due to in-season rainfall and humidity during the growing season, while, in 2018, low STB inoculum pressure was observed due to drought conditions. While STB responses across years were significantly correlated, relatively low correlations (typically of ~2016 and 2018, r = 0.15–0.37) were observed—likely due to differences in available moisture, prevailing temperature, pathogen population and trial design [90]. Broad sense heritability ranged between 0.14–0.22 (2016), 0.16–0.51 (2017), and 0.27–0.36 (2018). These are in line with previously estimated heritability for STB resistance [91,92,93].

Plant height and heading date can influence STB resistance as part of disease escape mechanisms [94,95,96,97]. In all datasets, plant height was negatively correlated with STB severity. This is because the lower leaves are the first to get infected at the start of the season, followed by vertical dispersal of pycnidiospores primarily due to rain splashes infecting leaves in the upper canopy. Thus, a greater internodal distance in the plants was associated with fewer STB symptoms. Similarly, flowering time impacts exposure to the STB epidemic as the uppermost leaves of later flowering varieties emerge later in the season when conditions are typically dryer, therefore acting as a disease escape mechanism. In general, late-flowering genotypes are preferred, as they can escape disease spread with plants appearing to have a resistant phenotype due to inoculum avoidance [35]. Therefore, residuals for STB were obtained after accounting for plant height and flowering time.

The selection of the founders is critical when developing MAGIC populations to ensure that the resulting population is genetically diverse and segregating for key traits. While higher yield per se is critical when selecting parents, the incorporation of other traits impacting yield such as disease resistance is also important. The founders of the “NIAB Elite MAGIC” population consist of eight genetically diverse winter wheat cultivars from UK and European wheat breeding programs, each contributing beneficial alleles for agronomic traits. STB response of the founders of the MAGIC population previously ranged between 5–7 on a scale of 1–9, where 1 = susceptible and 9 = resistant (https://ahdb.org.uk/rl; [98]). In our study, across datasets, all founders had depicted a range of STB scores that can be attributed to the vertical movement of the *Z. tritici* spores in the field, the extended length of exposure to the inoculum, the maximum inoculum load at the end of the season, and the plant growth stage correlating with onset of leaf senescence. In the study, two founders (Alchemy and Robigus), which were selected as MAGIC founders for various reasons, including disease resistance against multiple pathogens [49], displayed low disease scores. Additionally, Brompton and Claire displayed relatively low disease severity, suggesting other founders may also contribute to STB resistance in the population. Combinations of parental alleles at multiple loci across the genome have resulted in some of the MAGIC RILs having lower disease scores than the founders. Such transgressive segregation is a common feature of most traits segregating in MAGIC populations [53]

### 4.2. QTL Analysis and Comparison with Previously Reported STB QTL and Resistance Genes

We identified 25 QTL conferring resistances against STB. We also estimated the founder haplotype effects for resistance to STB for each QTL passing the permutated significance threshold (i.e., for the “strong” QTL) (Figure S4). Ten new QTL were detected in our study; however, these were identified using an arbitrary LOD threshold of 10 and are putative QTL with weak support. These ten QTL were located on chromosomes 1B (*QStb.teagasc-1B.1* and *QStb.teagasc-1B.2*), 1D (*QStb.teagasc-1D.1*), 2A (*QStb.teagasc-2A.2*), 3D (*QStb.teagasc-3D.1*), 4A (*QStb.teagasc-4A.1*), 4D (*QStb.teagasc-4D.1*), 6A (*QStb.teagasc-6A.2*), 6B (*QStb.teagasc-6B.1*), and 6D (*QStb.teagasc-6D.1*). The characterisation of novel QTL controlling STB resistance in the MAGIC population provides the knowledge from which markers could be developed to track beneficial alleles in wheat research and development programmes and to generate new allelic combinations across loci to provide improved resistance against STB. These sources of resistance can be further tested in multiple environments. Robust QTLs could then be exploited in combination with previously reported *Stb* genes and QTL via gene stacking.

In addition, we detected 15 QTL whose positions overlapped with previously published QTL conferring resistance to STB (detailed in Figure 3 and Table S5). Of these, four QTL (*QStb.teagasc-2D.1*, *QStb.teagasc-4B.1*, *QStb.teagasc-6D.2*, and *QStb.teagasc-7B.1*) were co-located with published adult plant STB resistance QTL identified under German field conditions [76], while nine QTL co-located with QTL from a single previously published study of field STB resistance in Switzerland [74]. Notably, *QStb.teagasc-7B.1* co-located with STB resistance QTL identified in both [74,76]. As these QTL appear to have been identified in more than one country, this suggests they may provide sources of durable resistance against different pathogen populations and prevailing conditions.

Five of the QTL detected in our study (QStb.teagasc-2D.1, QStb.teagasc-3A.1, QStb.teagasc-4B.1, QStb.teagasc-5A.1, and QStb.teagasc-7B.1) were found to co-locate with QTL (Qstb.B22-2D, Qstb.Z86-3A, Qstb.B22-3A, Qstb.Z86-4B.b, Qstb.Z86-5A, and Qstb.B22-7B.b) detected in a mapping study in two back backcross primary synthetic hexaploid wheat populations, in which they conferred resistance against natural STB infection at multiple locations in Germany [79]. Thus, the genomic region was considered to be potentially durable against STB. New and durable sources of genetic resistance against STB have previously been identified from synthetic hexaploid wheat, for instance, Stb5 on chromosome 7D [99], Stb8 on 7B [85], Stb16q on 3D [36], and Stb17 on 5A [36]. Although synthetic hexaploid wheat is a good source of diverse resistances against fungal diseases, breeders are reluctant to exploit these diverse sources of resistance due to the risk of linkage drag. However, diverse germplasm such as synthetic hexaploid wheat, landraces, and wild relatives can be backcrossed with elite cultivar and molecular markers used to address these difficulties and develop introgression lines. Introgression lines from wild relatives of wheat can also be used to deploy exotic alleles into elite genetic backgrounds. For instance, the variety Robigus, a commonly used parent in the European wheat pedigree [100] and also one of the eight founders of the “NIAB Elite MAGIC” population, contains putative *Triticum* *dicoccoides* introgressions and provides a durable source of STB resistance [49,68,101].

As *Stb.teagasc-7B.1* was the most significant locus identified in the MAGIC population, having been identified in seven of our 13 datasets, we consider this QTL in more detail here. *QStb.teagasc-7B.1* co-located with eight previously reported STB QTL (IWA814 [77], IWA3513 [77], AX-95223861 [76], *QTL_7B_1, and QTL_7B_2* [81], *QStb.B22-7B.b* [79], wsnp_JD_c646_966400 [80] and Interval_ID-17 [74]) and three *Stb* genes (*Stb8* [85], *Stb13* and *Stb14* [86]). We found resistance alleles at *QStb.teagasc-7B.1* to be conferred by the founders Alchemy, while alleles from the remaining five parents were associated with susceptibility (Figure S4). We also found flowering time QTL *QFt.teagasc-7B.1* in our study. The known vernalization response *VRN-B3* gene is located at 9.7 Mbp on chromosome 7B, which is far from the QTL (*QStb.teagasc-7B.1*) detected in our study. Furthermore, we used plant height and flowering time as covariates in the STB QTL analysis.

QTL conferring robust STB resistance under different field conditions has always been of particular interest for breeders. Here, we performed QTL analysis to identify genomic regions underpinning STB resistance in a MAGIC population using different mapping approaches. In our study, although trials were held over multiple years, the phenotypic data were from one location. Therefore, the data are likely insufficient to deduce the durability of these QTL and do not dissect line by location interaction. Testing in multiple locations will further increase understanding of the durability and underlying mechanisms of resistance. QTL detected by each single STB phenotype (i.e., time point and/or leaf combination) only explained relatively low proportions of the phenotypic variation in STB response (8.63–29.52%). This may also explain why only 12 of the QTL were detected across datasets, and the QTL have much larger intervals. The projection of QTL detected in this study on the physical map enabled us to determine if QTL had been identified previously. QTL detected across multiple studies could be the potential targets for durable STB resistance in breeding programs.

### 4.3. Breeding for Durable STB Resistance

The results presented here describe QTL for STB identified in the “NIAB Elite MAGIC” population under natural infection in a high-disease pressure environment. A single QTL can be introgressed using marker-assisted selection in breeding programs. However, combining a large number of QTL from multiple lines in a breeding program is challenging in terms of resources, epispastic interactions, and residual effects such as linkage drag and yield penalty. Therefore, resistance QTL may require prioritising for selection through successive cycles of breeding. Approaches such as genomic selection could be useful for quantitative resistance by estimating a line’s breeding value using genome-wide markers, and QTL identified here and in other studies can be used as fixed effects during genomic selection [75,102]. For instance, the prediction accuracy for STB resistance in wheat has been previously reported to have improved from 0.47 (without using QTL as fixed effects) to 0.62 (with using QTL as fixed effects) [75]. Ultimately, a pyramiding of qualitative and quantitative resistance has been highlighted as a strategy to extend the life of resistant cultivars [103], thereby providing multiple barriers against STB and reducing the number of asexual cycles in the field during the cropping season.

### 4.4. Conclusions

STB is one of the most devastating wheat diseases in north-western Europe as well as other wheat growing regions around the world with similar climatic conditions. In this study, we detected twenty-five genomic regions associated with STB resistance. While most of these QTL were in genomic regions previously linked with STB resistance, we identified new loci associated with resistance for potential use in the breeding of novel cultivars with increased resilience to STB.

## Figures and Tables

**Table 1:** Summary of experiments performed in this study at the adult plant stage for scoring septoria tritici blotch (STB) response across years and management applied.

Test Environment	Experiment Type	RILs Tested (n)	Data Collected	Time Points	Leaves	Datasets	Fungicide Applied ^1^
2016	Single row	1076	STB, plant height, days to flowering	T1 T2	flag, flag-1, flag-2	2016_T1_flag, 2016_T1_flag-1, 2016_T1_flag-2, 2016_T2_flag	Jenton
2017	6-row plot	192	STB, plant height, days to flowering	T1 T2	flag, flag-1, flag-2	2017_T1_flag, 2017_T1_flag-1, 2017_T1_flag-2, 2017_T2_flag, 2017_T2_flag-1, 2017_T2_flag-2,	Jenton ^2^
2018	Single row	388	STB	T1	flag, flag-1, flag-2	2018_T1_flag, 2018_T1_flag-1, 2018_T1_flag-2	Jenton/ Comet

^1^ The fungicides applied do not target STB. ^2^ The fungicide was applied twice; at the week of 3rd April and 24th May during the 2017 cropping season.

**Table 2:** Pearson correlation coefficients (r) between septoria tritici blotch (STB) and plant height and flowering time in the MAGIC population in field conditions under natural STB infection.

Datasets													
Year	2016				2017						2018		
Trait	T1_flag	T1_flag-1	T1_flag-2	T2_flag	T1_flag	T1_flag-1	T1_flag-2	T2_flag	T2_flag-1	T2_flag-2	flag	flag-1	flag-2
Flowering time	−0.17	−0.25	−0.29	−0.24	−0.19	−0.24	−0.29	−0.30	−0.17	−0.09	−0.14	−0.23	−0.23
Plant height	−0.28	−0.22	−0.20	−0.20	−0.23	−0.26	−0.26	−0.29	−0.20	−0.25	−0.22	−0.25	−0.23

Note: Disease assessment time points in each year (2016, 2017, and 2018) were labelled as T1 and T2, while the leaves assessed are labelled as flag, flag-1, and flag-2. All correlations were significant at *p* = 0.

**Table 3:** Pearson correlation coefficients (r) for septoria tritici blotch (STB) in the field under natural STB infection for all datasets across three field seasons. Best Linear Unbiased Estimates (BLUEs) calculated for each dataset after correction for plant height and flowering time were used.

	Datasets ^1,2,3^											
	2016				2017						2018	
	T1_flag	T1_flag-1	T1_flag-2	T2_flag	T1_flag	T1_flag-1	T1_flag-2	T2_flag	T2_flag-1	T2_flag-2	flag	flag-1
2016_T1_flag-1	0.54	1										
2016_T1_flag-2	0.49	0.71	1									
2016_T2_flag	0.34	0.52	0.51	1								
2017_T1_flag	0.38	0.47	0.44	0.42	1							
2017_T1_flag-1	0.43	0.66	0.5	0.48	0.65	1						
2017_T1_flag-2	0.35	0.59	0.5	0.44	0.62	0.82	1					
2017_T2_flag	0.4	0.56	0.52	0.55	0.55	0.7	0.61	1				
2017_T2_flag-1	0.42	0.61	0.6	0.56	0.58	0.77	0.69	0.84	1			
2017_T2_flag-2	0.32	0.42	0.47	0.38	0.32	0.55	0.51	0.59	0.7	1		
2018_flag	0.32	0.35	0.41	0.4	0.39	0.4	0.35	0.51	0.51	0.41	1	
2018_flag-1	0.34	0.47	0.51	0.48	0.34	0.44	0.49	0.59	0.53	0.44	0.69	1
2018_flag-2	0.26	0.48	0.5	0.47	0.29	0.4	0.46	0.55	0.51	0.44	0.62	0.83

^1^ Each dataset name (e.g., 2016_T1_flag) comprise year (i.e., 2016, 2017, and 2018), disease assessment time points (i.e., T1 and T2), and leaves (i.e., flag, flag-1, and flag-2) indicated. ^2^ All the values were significant at p-values of 0.0. ^3^ Cell colour changes from red to green with increased correlation coefficients.

**Table 4:** Summary of percentage septoria tritici blotch (STB) infection in the MAGIC population, along with STB scores for the eight founders across all datasets. The adjusted means were logit (p) back-transformed to a 0–100 percentage scale. The data shown includes the total number of lines, range, and population mean in each dataset along with the mean disease severity (%) of the founder in each dataset.

Dataset ^1^	MAGIC Population			Founders							
	Total Lines	Range	Mean	Alchemy	Brompton	Claire	Hereward	Rialto	Robigus	Soissons	Xi19
	*(n)*	(min–max)	(µ)								
2016_T1_flag	1072	0.11–17.09	1.75	- ^2^	1.17	-	5.22	-	1.03	4.57	2.18
2016_T1_flag-1	1072	0.58–30.85	6.04	-	7.97	2.30	3.77	2.48	2.91	6.12	6.90
2016_T1_flag-2	1072	3.032–85.97	20.19	-	26.34	12.58	21.38	9.54	20.47	23.52	18.43
2016_T2_flag	1072	0.68–86.34	17.52	-	15.85	16.31	19.01	34.24	22.56	38.52	12.19
2017_T1_flag	191	0.35–8.98	2.42	0.75	2.50	1.75	2.12	3.99	0.75	2.53	1.77
2017_T1_flag-1	191	0.71–33.04	6.55	4.97	4.78	2.65	4.61	14.96	2.38	11.16	5.95
2017_T1_flag-2	191	2.63–65.69	17.21	9.86	9.95	11.62	9.70	61.04	8.55	35.01	18.11
2017_T2_flag	191	3.42–79.96	16.51	6.71	23.35	7.55	11.12	15.34	7.37	43.35	17.23
2017_T2_flag-1	191	5.758–95.00	46.51	21.25	36.24	29.40	53.87	51.35	33.18	88.30	74.74
2017_T2_flag-2	191	13.51–92.90	68.91	63.98	67.01	79.29	85.90	55.70	37.99	92.79	84.23
2018_flag	374	0.26–47.1	2.47	-	2.92	0.76	1.29	2.30	1.14	-	-
2018_flag-1	374	0.67–79.99	8.63	-	6.34	1.50	2.85	11.34	3.84	-	-
2018_flag-2	374	1.22–87.28	34.31	-	18.67	7.50	28.13	61.66	21.88	-	-

^1^ Each dataset name (e.g., 2016_T1_flag) comprise year (i.e., 2016, 2017, and 2018), disease assessment time points (i.e., T1 and T2), and leaves (i.e., flag, flag-1, and flag-2). ^2^ No data were available.

**Table 5:** Summary of the septoria tritici blotch (STB) resistance QTL identified using Haley-Knott regression method in the MAGIC population grown in the field under natural STB infection.

QTL Name	Dataset	Chr.	Interval (cM) ^1^	Interval (Mbp) ^2^	Flanking Markers	Peak Marker	Pos (cM)	Phy.pos (Mbp)	LOD ^4^	LOD thr ^5^	R ^2^ (%)
*QStb.teagasc-1A.1*	2017_T1_flag-1	1A	66.54–106.72	224.83–482.97	Ex_c21450_396- RFL_Contig3203_1971	BobWhite_c38865_319	95.09	465.28	11.19	13.97	26.42
*QStb.teagasc-1B.1*	2018_T1_flag-1	1B	46.87–69.6	53.25–240.48	Excalibur_c46902_92- RFL_Contig2852_1839	Kukri_c18006_1568	56.55	563.11	10.74	12.46	13.8
*QStb.teagasc-1B.2*	2017_T2_flag	1B	283.53–299.76	648.45–662.72	BS00043666_51- BS00078414_51	Kukri_rep_c102102_273^3^	291.21	563.11	10.19	15.4	24.36
*QStb.teagasc-1D.1*	2017_T2_flag	1D	103.8–131.34	423.25–490.6	Excalibur_c23473_451- Excalibur_c6476_811	Excalibur_c6476_811	131.34	460.59	10.07	15.4	24.13
*QStb.teagasc-2A.1*	2018_T1_flag-1	2A	139.25–150.46	605.18–677.53	BS00049644_51- IAAV4015	RAC875_c38018_278	144.81	639.98	10.31	12.46	13.28
	2016_T2_flag	2A	142.77–150.46	615.29–677.53	BS00062679_51- IAAV4015	BS00022241_51	146.83	663.32	12.38	14.03	9.18
	2018_T1_flag-2	2A	142.77–150.46	615.29–677.53	BS00062679_51- IAAV4015	BS00022241_51	146.83	663.32	10.83	12.13	13.91
*QStb.teagasc-2A.2*	2016_T1_flag-2	2A	249.27–259.39	765.26–770.02	BS00107649_51- BS00064055_51	BS00064055_51	259.39	765.26	10.9	14.28	8.13
*QStb.teagasc-2B.1*	2017_T1_flag-2	2B	216.09–259.25	115.11–553.62	RFL_Contig4718_1323- IACX5941	Kukri_c693_87	232.95	636.11	10.04	13.16	24.07
	2016_T2_flag	2B	224.28–366.34	272.8^3^–595.13	BS00022800_51- tplb0042o21_419	Excalibur_c105074_293	235.52	109.53	10.47	14.03	7.82
	2016_T1_flag-2	2B	317.29–366.34	272.8^3^–774.95	RFL_Contig2751_1562- tplb0042o21_419	BS00067878_51	336.02	784.62	11.93	14.28	8.86
*QStb.teagasc-2D.1*	2017_T2_flag	2D	0.5–21.94	9.34^3^ –642.87	tplb0060e06_1793- D_contig17313_245	BS00063251_51	15.33	-	11.43	15.4	26.91
*QStb.teagasc-3A.1*	2016_T2_flag	3A	1.51–107.22	137.58^3^–336.78	RAC875_c3141_214- BS00030876_51	RFL_Contig4403_1034	98.98	176.55	10.01	14.03	7.49
	2017_T1_flag-1	3A	22.81–176.22	13.41–611.7	BS00104401_51- wsnp_Ex_c26887_36107413	Tdurum_contig10307_375	166.27	538.01	11.13	13.97	26.3
	2017_T2_flag	3A	139.3–308.8	543.18–749.33	Tdurum_contig59531_914- Tdurum_contig27982_568	wsnp_Ex_c1335_2556442	163.97	532.33	11.45	15.4	26.93
	2017_T2_flag-1	3A	139.3–174.7	543.18–616.49	Tdurum_contig59531_914- BS00056089_51	wsnp_Ex_c1335_2556442	163.97	532.33	10.24	13.98	24.48
*QStb.teagasc-3B.1*	2016_T1_flag-1	3B	49.33–60.59	24.94–39.46	Tdurum_contig22897_107- Excalibur_c79902_439	Excalibur_c24391_321	57.06	31.1	11.55	12.39	8.59
	2016_T1_flag-2	3B	49.33–60.59	24.94–39.46	Tdurum_contig22897_107- Excalibur_c79902_439	Excalibur_c24391_321	57.06	31.1	11.22	14.28	8.36
	**2016_T2_flag**	**3B**	**49.33–63.63**	**24.94–42.34**	**Tdurum_contig22897_107-** **BS00022242_51**	**Excalibur_c24391_321**	**57.06**	**31.1**	**14.51**	**14.03**	**10.68**
	2016_T1_flag	3B	57.06–95.19	31.11–145.31	Excalibur_c24391_321- IACX971	BS00073732_51	87.63	67.92	10.36	12.39	7.74
*QStb.teagasc-3D.1*	2016_T1_flag-2	3D	100.88–119.23	274.49–524.87	Ku_c2845_342- IAAV5136	Ku_c6080_1667	107.27	488.85	11.07	14.28	8.25
*QStb.teagasc-4A.1*	2017_T1_flag-1	4A	33.88–74.98	16.19–68.56	wsnp_Ex_c28429_37553452- wsnp_Ex_c10390_17007929	BS00110459_51	66.77	41.94	11.2	13.97	26.44
	2016_T1_flag-1	4A	55.12–164.01	30.29–632.86	Kukri_c16916_1073- BS00091752_51	BS00108849_51	132.74	617.28	10.62	12.39	7.93
	2017_T2_flag	4A	91.12–103.23	583.95–597.69	BS00010339_51- BS00077716_51	BS00040648_51	96.69	594.66	12.09	15.4	28.21
*QStb.teagasc-4B.1*	2017_T2_flag	4B	0–173.22	5.47–665.6	BS00039936_51- BS00009342_51	wsnp_BF482960B_Ta_1_4	47.64	289.54	12.24	15.4	28.51
	2017_T1_flag-2	4B	124.46–158.5	575.6–620.06	BobWhite_c11005_236- BobWhite_c27751_206	Tdurum_contig93160_155	148.22	576.2	11.48	13.16	27.01
	2017_T1_flag-1	4B	125.98–182.54	590.44–637.39	Ku_c48056_436- wsnp_Ku_c12503_20174234	RAC875_c1918_101	152.89	636.77	10.83	13.97	25.69
	2017_T2_flag-1	4B	148.22–179.45	576.2–645.3	Tdurum_contig93160_155- RAC875_c24515_602	Ra_c10455_3226	166.59	590.44	12.19	13.98	28.4
	2016_T2_flag	4B	189.64–209.52	637.39–660.47	BS00035426_51- IACX7540	RAC875_c87897_333	196.07	650.94	10.2	14.03	7.62
*QStb.teagasc-4D.1*	2016_T1_flag-2	4D	0–110.72	6.6–481.61^3^	BS00054978_51- IAAV5607	BS00064176_51	104.49	483.29	10.85	14.28	8.09
	2016_T2_flag	4D	0–9.87	1.24–6.6	BS00054978_51- Excalibur_c26088_184	BS00099053_51	4.1	3.61	10.32	14.03	7.72
	2017_T2_flag	4D	9.87–40.11	1.24–25.99	Excalibur_c26088_184- RAC875_rep_c105718_304	RAC875_c1673_663	32.24	16.59	11	15.4	26.03
*QStb.teagasc-5A.1*	2017_T1_flag-1	5A	0–138.95	0.64–494.87	BobWhite_c7114_237- wsnp_Ku_c35386_44598937	Excalibur_rep_c104815_1181	6.08	3.4	10.43	13.97	24.86
	2017_T2_flag-1	5A	0–138.95	0.64–494.87	BobWhite_c7114_237- wsnp_Ku_c35386_44598937	BS00015653_51	98.27	474.5	10.16	13.98	24.32
	2017_T1_flag	5A	41.26–94.7	29.51–475.47	Excalibur_rep_c90275_262- tplb0057m23_716	BS00066499_51	82.55	-	10.12	12.47	24.24
*QStb.teagasc-5B.1*	2016_T1_flag-2	5B	1.27–282.07	17.97–690.7	BS00015136_51- BS00092233_51	Kukri_c60322_490	6.88	19.44	10.62	14.28	7.93
	2018_T1_flag-2	5B	184–202.43	589.12	RAC875_c30867_515-IAAV4388	BS00061414_51	189.1	580.7	10.07	12.13	13
	2017_T1_flag-1	5B	230.67–261.48	647.94	BobWhite_c12883_272- Excalibur_c9563_1157	BS00062972_51	258.92	670.54	10.66	13.97	25.34
	2017_T2_flag	5B	253.87–261.48	668.47	RAC875_c45135_184- Excalibur_c9563_1157	Excalibur_c17489_804	258.92	670.82	12.02	15.4	28.08
*QStb.teagasc-5D.1*	2016_T1_flag-2	5D	12.57–49.43	47.02–389.93	BS00003975_51- BobWhite_c7263_337	IACX2960	28.11	347.48	11.92	14.28	8.85
*QStb.teagasc-6A.1*	2018_T1_flag-1	6A	21.25–65.6	6.2– 22.44	Ra_c22493_190-BobWhite_c13839_135	Excalibur_c7044_243	56.01	18.71	10.79	12.46	13.86
	**2016_T1_flag**	**6A**	**56.01–70.12**	**18.71–27.5**	**Excalibur_c7044_243-** **RAC875_c18659_402**	**BS00022951_51**	**61.07**	**18.71**	**12.77**	**12.39**	**9.45**
	2016_T2_flag	6A	88.28–106.44	32.66–65.07	wsnp_Ex_c31149_39976103- BS00021999_51	Kukri_c27958_334	96.41	51.51	10.89	14.03	8.12
	2017_T1_flag	6A	88.28–106.95	32.66–70.4	wsnp_Ex_c31149_39976103- wsnp_Ku_c18534_27848426	Jagger_c2853_75	93.31	51.4	10.5	12.47	25.02
	2016_T1_flag-2	6A	103.35–137.51	51.95–309.86	Tdurum_contig50698_601- BS00065309_51	BS00026558_51	127.92	94.2	11.31	14.28	8.42
*QStb.teagasc-6A.2*	2017_T2_flag-1	6A	173.36–191.33	534.67–580.19	BS00028263_51- wsnp_BE495143A_Ta_2_2	BS00023893_51	176.94	557.93	11.31	13.98	26.66
*QStb.teagasc-6B.1*	2016_T1_flag-2	6B	35.81–57.49	21.41–48.35	wsnp_Ex_c702_1382859- wsnp_Ex_c13352_21044607	BS00090073_51	51.22	41.97	11.12	14.28	8.28
*QStb.teagasc-6B.2*	2016_T2_flag	6B	92.34–109.98	152.09–168.47^3^	BS00063608_51- BobWhite_c686_387	wsnp_Ex_c34011_42398362	104.92	462.13	10.3	14.03	7.7
*QStb.teagasc-6B.3*	**2018_T1_flag**	**6B**	**128.13–149.57**	**193.46**	**Kukri_c23433_416-** **BobWhite_c14575_323**	**BS00048295_51**	**133.69**	**513.69**	**14.3**	**13.55**	**17.94**
	2018_T1_flag-1	6B	128.13–149.57	193.46	Kukri_c23433_416- BobWhite_c14575_323	BS00048293_51	134.69	513.69	12.38	12.46	15.74
	2018_T1_flag-2	6B	129.66–144.9	465.68–521.03	RFL_Contig3609_1678- wsnp_Ku_c24981_34948114	BS00035381_51	141.88	542.22	11.4	12.13	14.59
*QStb.teagasc-6D.1*	2017_T2_flag-1	6D	25.79–102.17	1.77–315.6	Excalibur_c10358_1800- IACX10982	BS00021970_51	53.79	24	12.2	13.98	28.43
*QStb.teagasc-6D.2*	2016_T1_flag-2	6D	196.75–215	462.2–472.79	BobWhite_c34798_184- BS00070856_51	wsnp_Ex_c13188_20825019	202.62	464.71	10.15	14.28	7.59
*QStb.teagasc-7B.1*	2016_T2_flag	7B	73.93–242.34	106.34–625.52	BobWhite_c14812_828- wsnp_Ex_c64848_63486322	BobWhite_c24067_519^3^	213.89	713.36	11.9	14.03	8.84
	2016_T1_flag-2	7B	109.14–239.29	472.33–732.15	Ra_c3470_1551- Kukri_c40149_436	Tdurum_contig30163_105^3^	185.27	701.63	11.6	14.28	8.63
	2017_T1_flag	7B	190.34–225.24	701.34–718.36	RAC875_rep_c78007_425- BS00022162_51	Tdurum_contig67161_99	203.23	703.3	10.46	12.47	24.93
	2017_T2_flag	7B	193.88–216.43	701.34–712.73	RAC875_c41938_471- BS00066124_51	Tdurum_contig13268_1067	194.89	708.95	12.76	15.4	29.52
	2017_T2_flag-1	7B	193.88–239.29	701.34–732.15	RAC875_c41938_471- Kukri_c40149_436	Tdurum_contig81587_90	195.39	706.81	11.55	13.98	27.14
	2017_T1_flag-1	7B	208.8–240.81	706.81–727.63	Tdurum_contig61856_900- wsnp_Ex_c4484_8065800	BobWhite_c2892_167	233.61	721.21	10.06	13.97	24.1
	**2016_T1_flag-1**	**7B**	**212.37–250.49**	**713.66–732.65**	**RAC875_c39269_312-** **wsnp_Ex_c32905_41484291**	**BobWhite_c2892_167**	**233.61**	**721.21**	**13.8**	**12.39**	**10.18**

^1^. 1.5-LOD support interval calculated to identify multiple peaks; reported the lowest and the highest position at a 95% confidence interval. ^2^. The physical map position for each quantitative trait loci (QTL) (flanking and peak markers) was obtained from the wheat Chinese Spring IWGSC RefSeq v1.0genome assembly. ^3^. Where the first BLASTn hit from the physical map (IWGSC RefSeq v1.0) is located on one of the homoeologous chromosomes compared to its true genetically mapped location on the MAGIC genetic map [68], and where the BLASTn hit to the correct homoeologous chromosome has an equal or very similar e-value to the first hit, then chromosome and Mbp position used here is that for the chromosome to which the SNP was genetically mapped in [68]. ^4^. Logarithm of odds (LOD). ^5^. LOD permutation significance threshold (α = 0.05). 6. QTL information in bold is permutation significant at α = 0.05, while others are significant at the arbitrary threshold (LOD = 10.0).

## Supplementary Materials

The following are available online at https://www.mdpi.com/2073-4425/11/8/887/s1. The genotypic information including genotypic data, genetic map, and pedigree of the MAGIC population used in this study are publicly available at https://www.niab.com/research/research-projects/resources. The raw phenotypic data for the MAGIC population used in this study are publicly available at https://figshare.com/account/home#/projects/78765. The following are available online at https://www.mdpi.com/2073-4425/11/8/887/s1. **Figure S1**. Density distribution (blue dotted line) and normal fit (red dotted line) plot of each dataset. The title of each graph such as “2016_T1_flag” represents year (2016, 2017, and 2018), time points (T1 and T2), and different leaves (flag, flag-1, and flag-2). The prefix “logit” is added to logit-transformed phenotypic data. In 2018, plant height and days to flowering data were used as surrogate from 2016 dataset. **Figure S2**. Density distribution (blue dotted line) and normal fit (red dotted line) plot of each dataset after correcting for plant height and flowering time. The title of each graph such as “2016_T1_flag” represents year (2016, 2017, and 2018), time points (T1 and T2), and different leaves (flag, flag-1, and flag-2). **Figure S3**. LOD scores from QTL mapping for septoria tritici blotch (STB) resistance in the NIAB Elite MAGIC population displayed for each dataset. Each of the individual datasets contains the LOD scores of the genome scan using the Haley-Knott regression, linear mixed model (LMM), and leave one chromosome out (LOCO) model. The “red” line indicates 5% level of genome-wide significance threshold based on permutation test, while “blue dotted line” represents arbitrary threshold for all the datasets (LOD = 10.0), determining weak QTL. **Figure S4**. Estimated allele effects at different QTL for STB resistance in the NIAB Elite MAGIC population across the dataset. In each of the individual dataset, the upper panel represents BLUPs of the eight haplotype founder effects in the additive model, along with the LOD curve on the respective chromosomes. The title of each figure represents QTL and the dataset in which it was detected. The LOD scores of the genome scans represented here were determined by Haley-Knott regression. **Table S1**. Phenotypic data for STB, flowering time and plant height for the NIAB Elite MAGIC population collected from different datasets. **Table S2**. Summary of LOD scores for QTL mapping of NIAB Elite MAGIC population across different datasets. LOD scores were obtained from three different methods by Haley-Knott (hk) regression, a linear mixed model (lm) using standard kinship matrix, and a linear mixed model using leave one chromosome out (LOCO) kinship matrix. **Table S3**. Summary of the flowering time and plant height QTL identified using the Haley-Knott regression method in the NIAB Elite MAGIC population grown in the field under natural STB infection. QTL were compared with flowering time and plant height data for NIAB Elite MAGIC population from the previously published study by Scutari et al. [84]. **Table S4**. Functional annotation of peak marker of the septoria tritici blotch (STB) resistance QTL identified in this study. **Table S5**. Comparison of physical positions of the QTL identified in the present study with those reported previously.
