# Supplementary material for: Genetic Analysis Using a Multi-Parent Wheat Population Identifies Novel Sources of Septoria Tritici Blotch Resistance
Source: Genes (Basel). 2020 Aug 4;11(8):887. doi: 10.3390/genes11080887 (PMC7465482; doi:10.3390/genes11080887)
Supplement: Supplementary file 1 [file genes-11-00887-s001.zip › Figure S1.pdf]

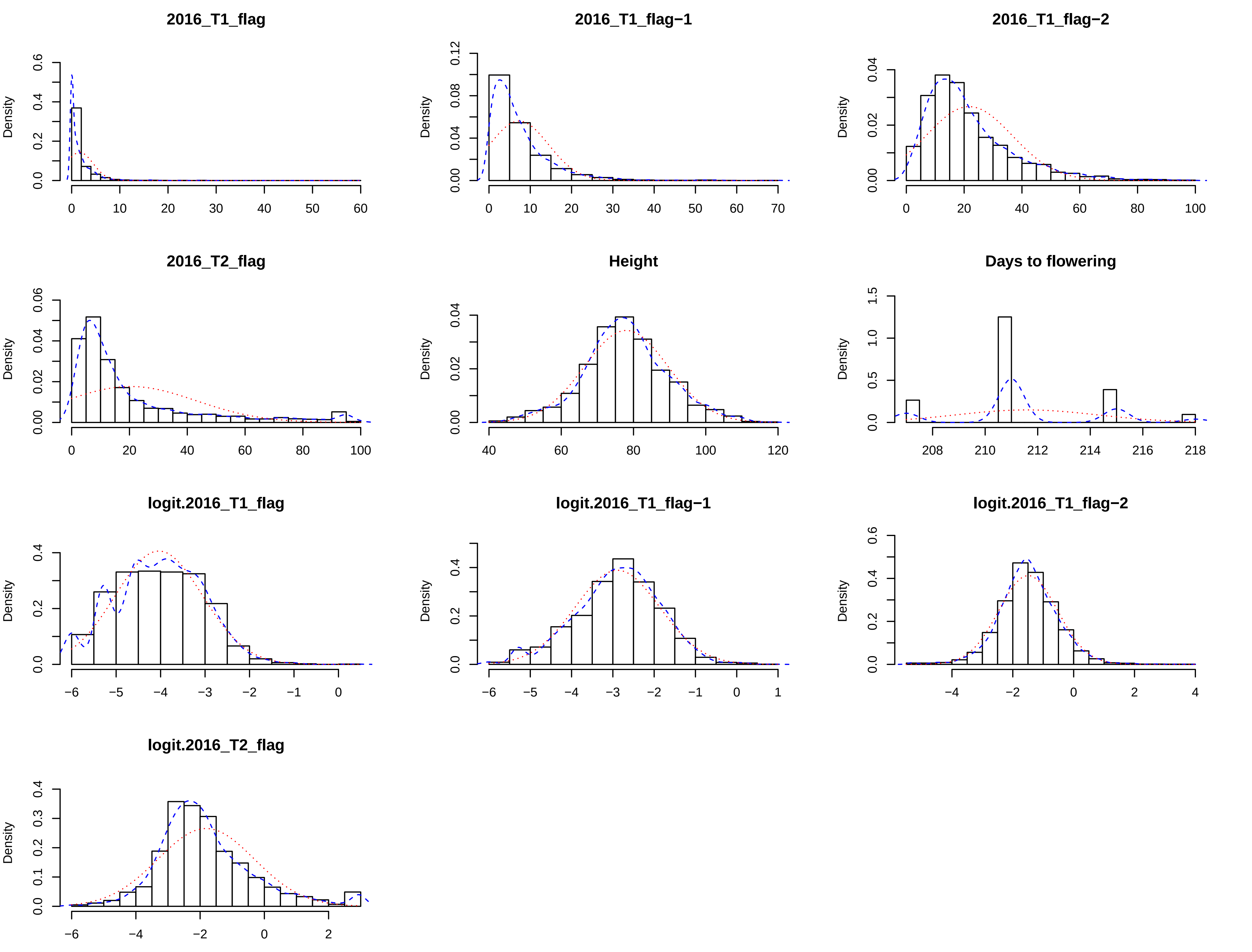
2016_T1_flag
2016_T1_flag−1
2016_T1_flag−2
2016_T2_flag
Height
Days to flowering
logit.2016_T1_flag
logit.2016_T1_flag−1
logit.2016_T1_flag−2
logit.2016_T2_flag
Density

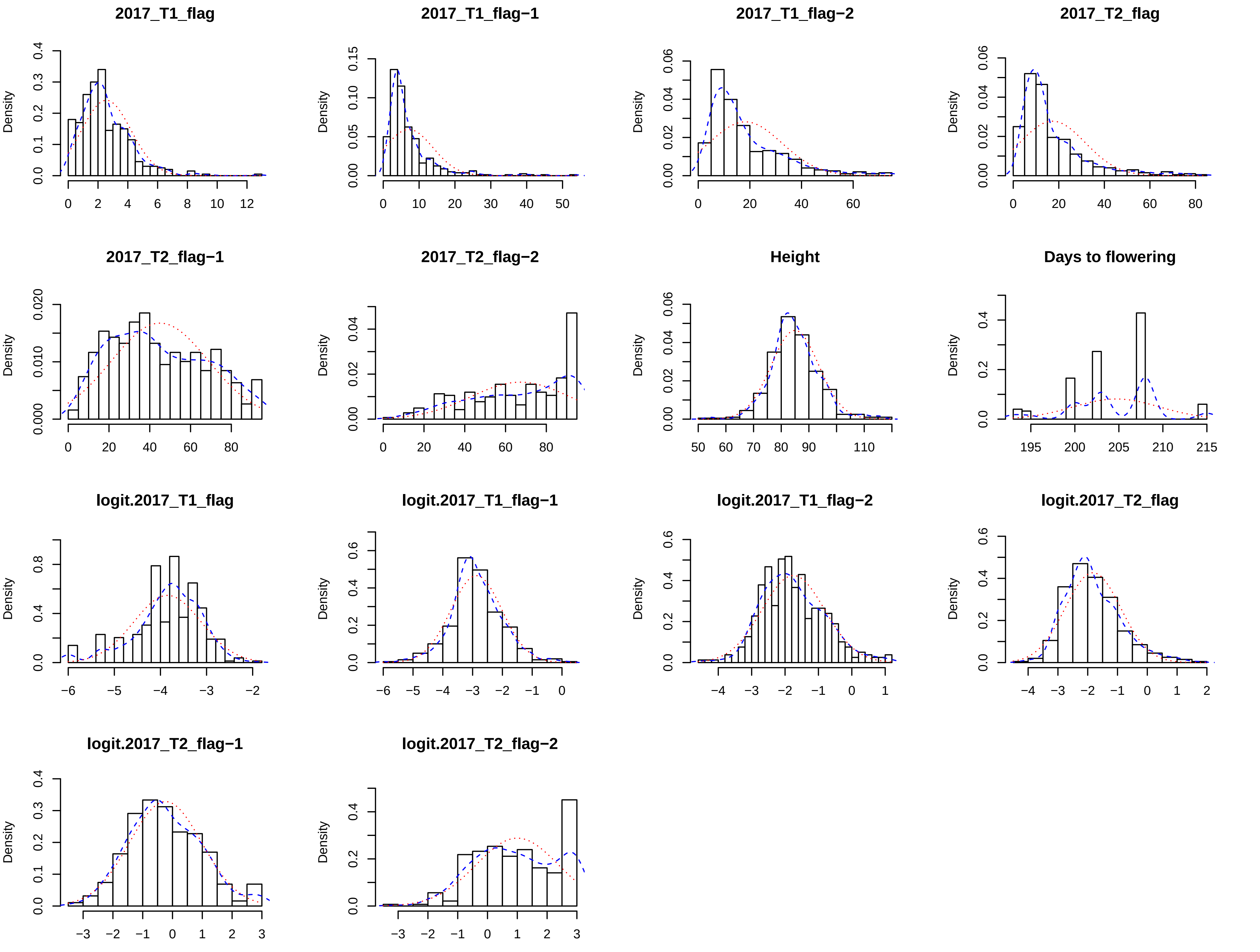

2017_T1_flag
2017_T1_flag−1
2017_T1_flag−2
2017_T2_flag
2017_T2_flag−1
2017_T2_flag−2
Height
Days to flowering
logit.2017_T1_flag
logit.2017_T1_flag−1
logit.2017_T1_flag−2
logit.2017_T2_flag
logit.2017_T2_flag−1
logit.2017_T2_flag−2
Density

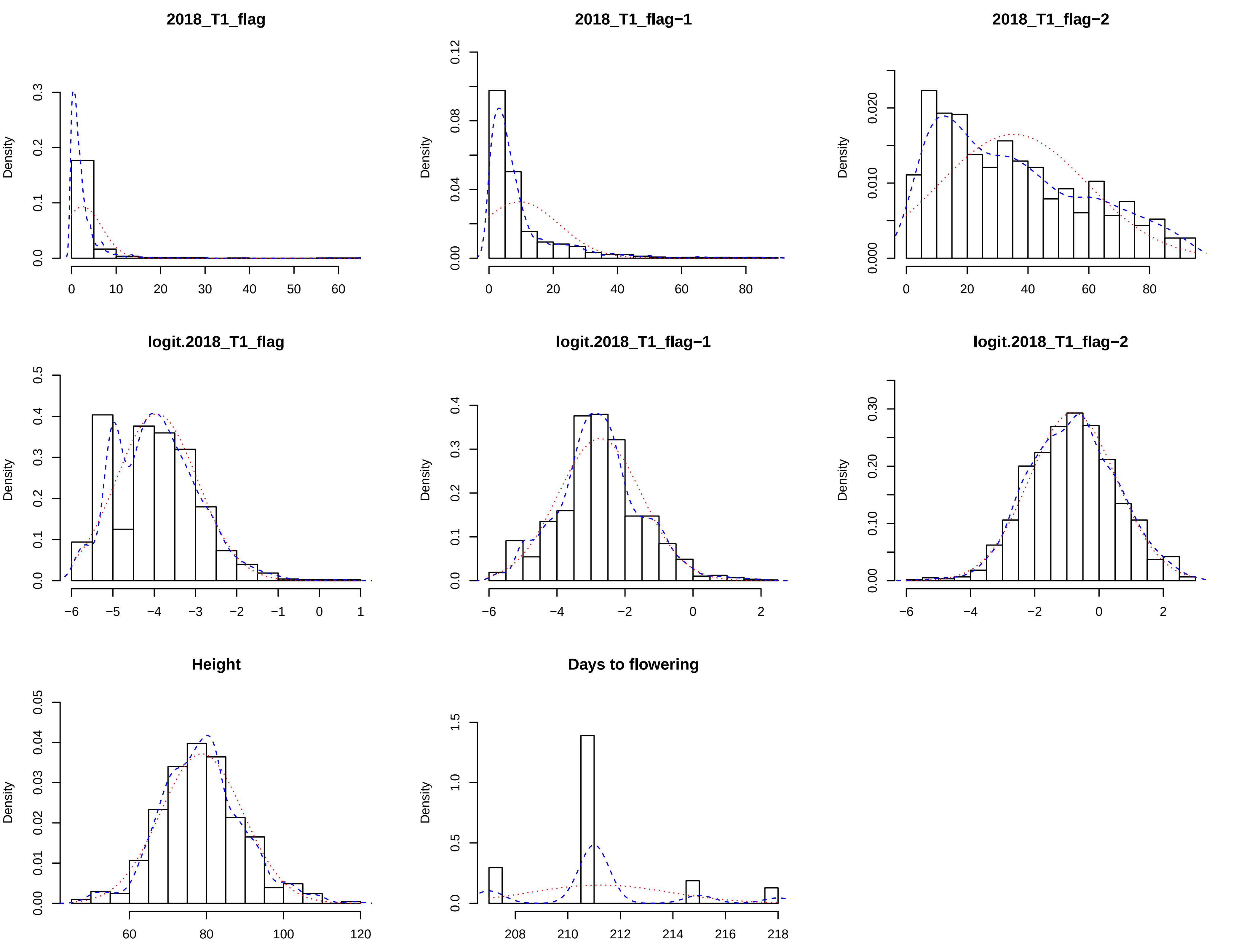
2018_T1_flag
2018_T1_flag−1
2018_T1_flag−2
logit.2018_T1_flag
logit.2018_T1_flag−1
logit.2018_T1_flag−2
Height
Days to flowering
Density
0.0 0.1 0.2 0.3
0 10 20 30 40 50 60
0.00 0.04 0.08 0.12
0 20 40 60 80
0.000 0.010 0.020
0.0 0.1 0.2 0.3 0.4 0.5
−6 −5 −4 −3 −2 −1 0 1
0.0 0.1 0.2 0.3 0.4
−6 −4 −2 0 2
0.00 0.10 0.20 0.30
0.00 0.01 0.02 0.03 0.04 0.05
60 80 100 120
0.0 0.5 1.0 1.5
208 210 212 214 216 218

Figure S1. Density distribution (blue dotted line) and normal fit (red dotted line) plot of each dataset. The title of each graph such as "2016_T1_flag" represents year (2016, 2017, and 2018), time points (T1 and T2) and different leaves (flag, flag-1 and flag-2). The prefix "logit" is added to logit-transformed phenotypic data. In 2018, plant height and days to flowering data were used as surrogate from 2016 dataset.
