# Supplementary material for: Genetic Analysis Using a Multi-Parent Wheat Population Identifies Novel Sources of Septoria Tritici Blotch Resistance
Source: Genes (Basel). 2020 Aug 4;11(8):887. doi: 10.3390/genes11080887 (PMC7465482; doi:10.3390/genes11080887)
Supplement: Supplementary file 1 [file genes-11-00887-s001.zip › Figure S2.docx]

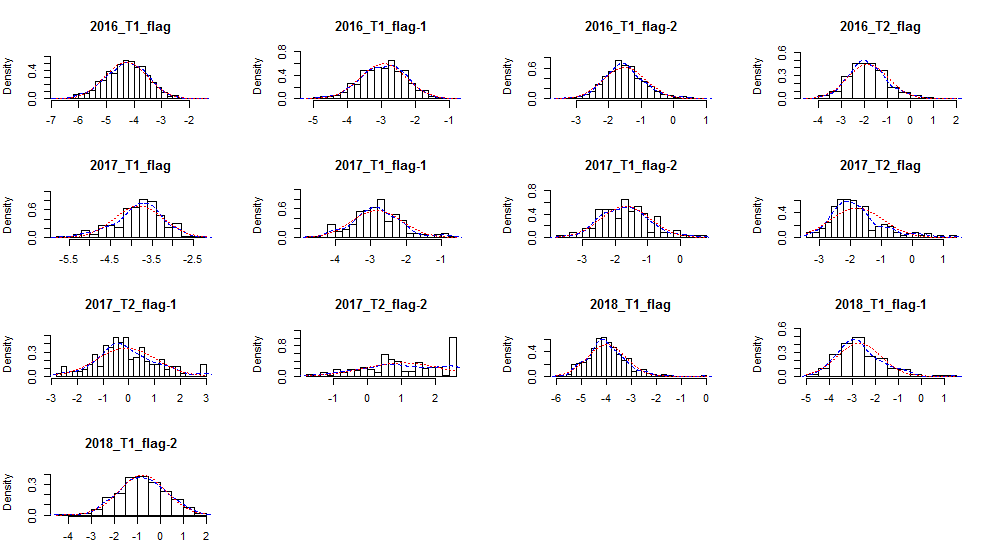


**Figure S2.** Density distribution (blue dotted line) and normal fit (red dotted line) plot of adjusted STB scores in all datasets. The title of each graph such as “2016_T1_flag” represents year (2016, 2017, and 2018), time points (T1 and T2) and different leaves (flag, flag-1 and flag-2).
