# Supplementary material for: Genetic Analysis Using a Multi-Parent Wheat Population Identifies Novel Sources of Septoria Tritici Blotch Resistance
Source: Genes (Basel). 2020 Aug 4;11(8):887. doi: 10.3390/genes11080887 (PMC7465482; doi:10.3390/genes11080887)
Supplement: Supplementary file 1 [file genes-11-00887-s001.zip › Figure S3.pdf]

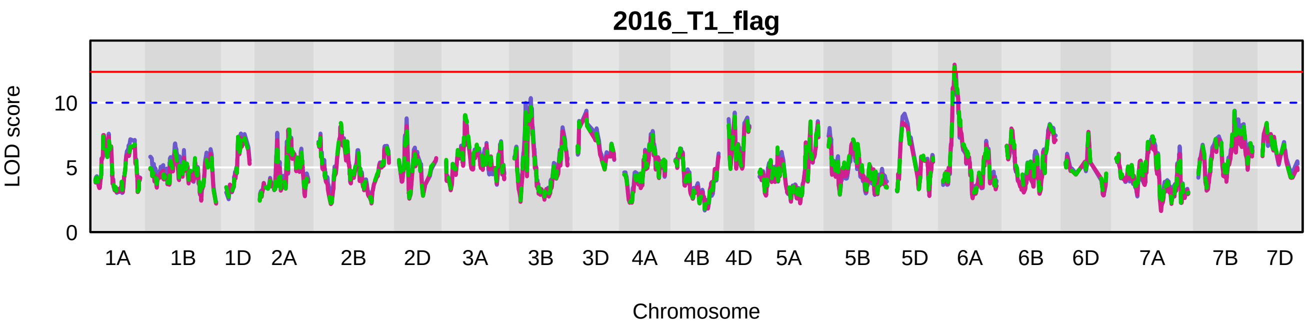
2016_T1_flag
LOD score
0
5
10
1A
1B
1D
2A
2B
2D
3A
3B
3D
4A
4B
4D
5A
5B
5D
6A
6B
6D
7A
7B
7D
Chromosome

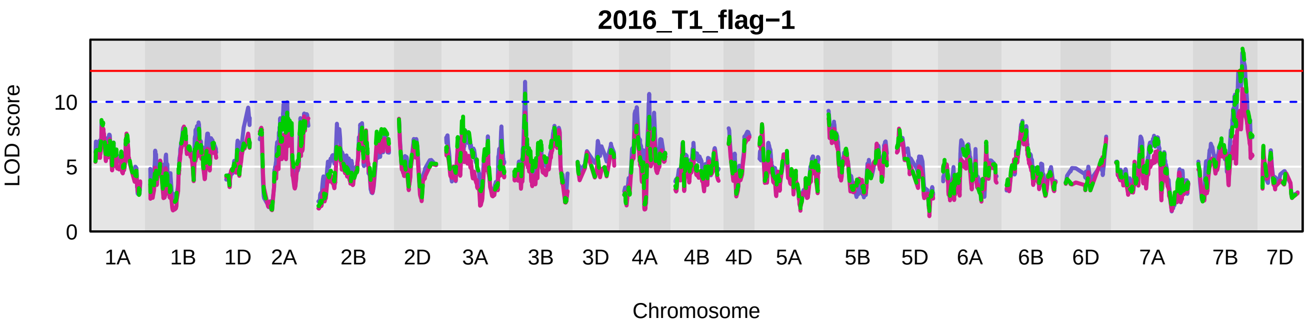
2016_T1_flag−1
LOD score
0
5
10
1A
1B
1D
2A
2B
2D
3A
3B
3D
4A
4B
4D
5A
5B
5D
6A
6B
6D
7A
7B
7D
Chromosome

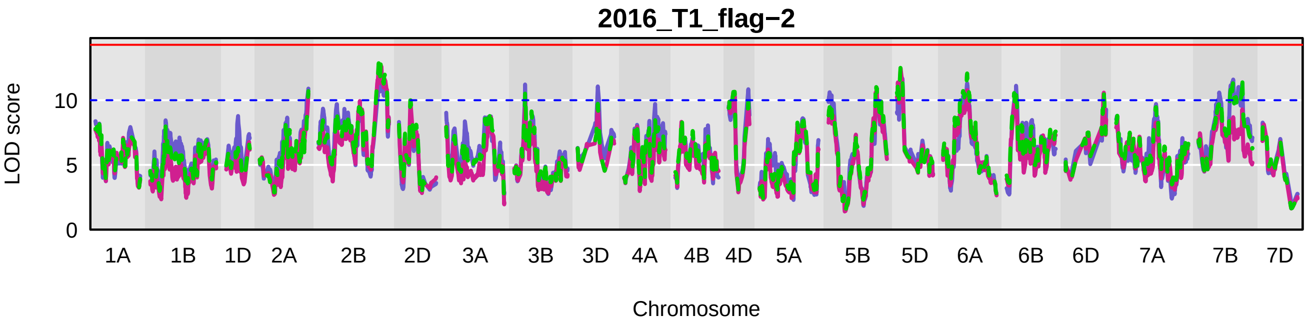
2016_T1_flag−2
LOD score
0
5
10
1A
1B
1D
2A
2B
2D
3A
3B
3D
4A
4B
4D
5A
5B
5D
6A
6B
6D
7A
7B
7D
Chromosome

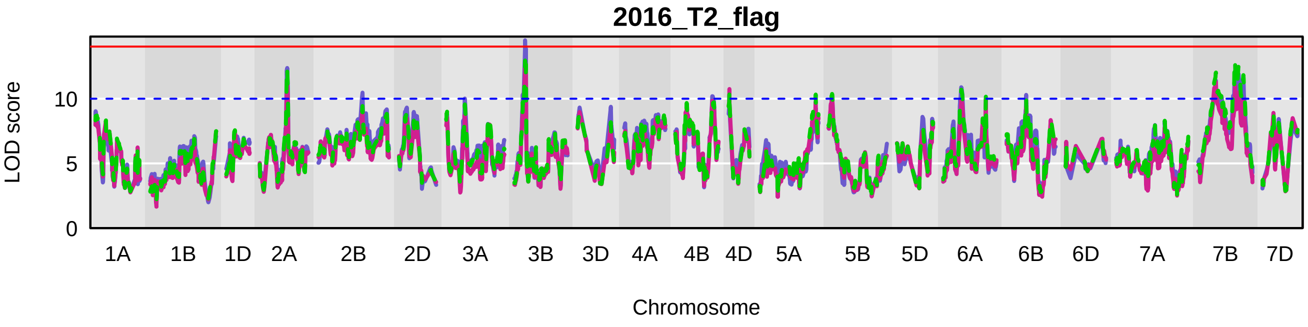
2016_T2_flag
LOD score
0
5
10
1A
1B
1D
2A
2B
2D
3A
3B
3D
4A
4B
4D
5A
5B
5D
6A
6B
6D
7A
7B
7D
Chromosome

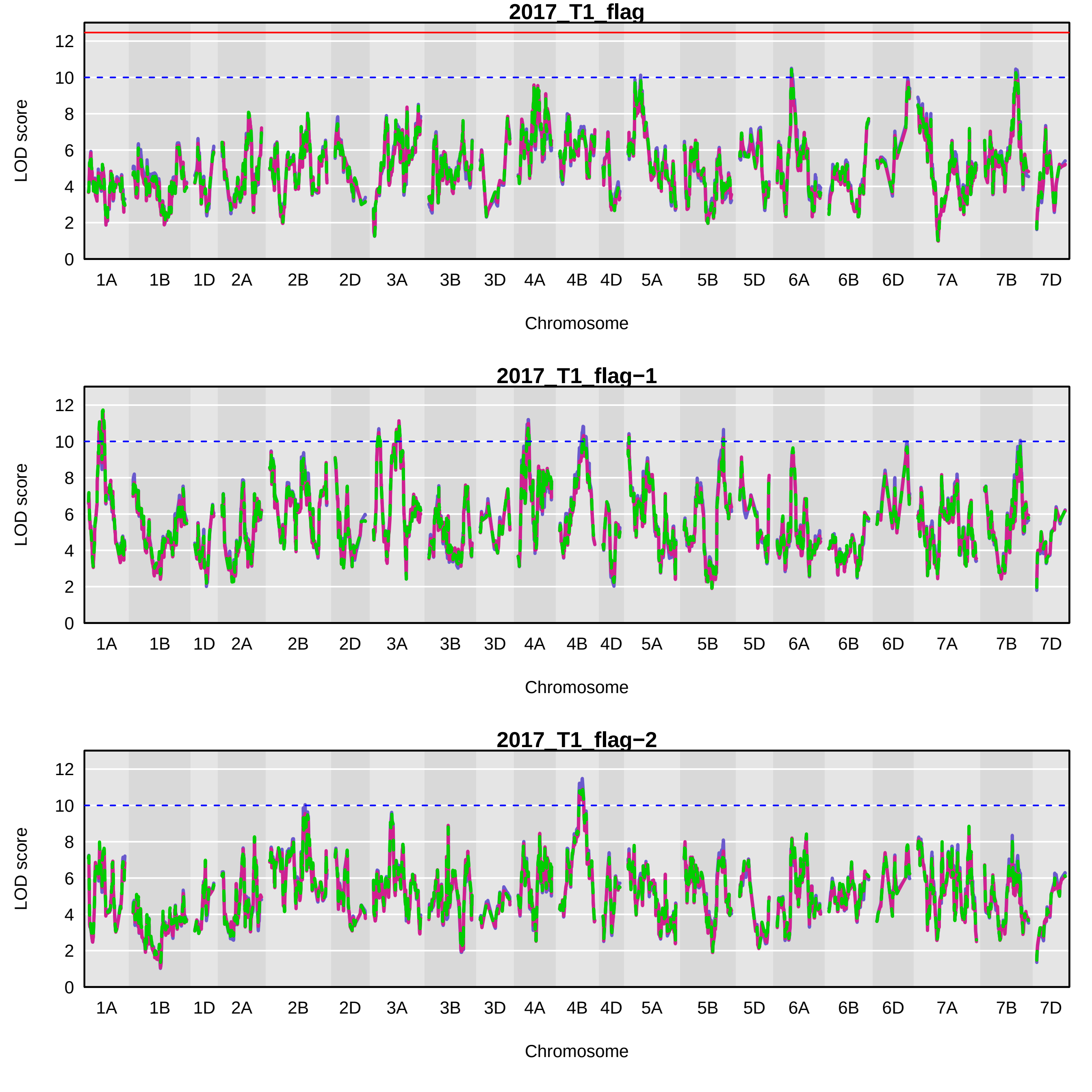

2017_T1_flag
LOD score
0
2
4
6
8
10
12
1A
1B
1D
2A
2B
2D
3A
3B
3D
4A
4B
4D
5A
5B
5D
6A
6B
6D
7A
7B
7D
Chromosome
2017_T1_flag−1
LOD score
Chromosome
2017_T1_flag−2
LOD score
Chromosome

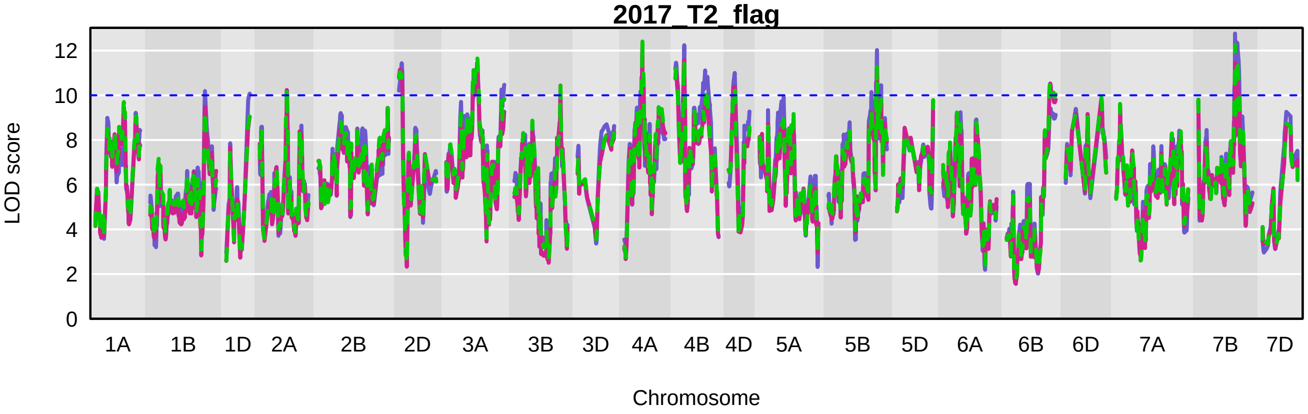
2017_T2_flag
LOD score
0
2
4
6
8
10
12
1A 1B 1D 2A 2B 2D 3A 3B 3D 4A 4B 4D 5A 5B 5D 6A 6B 6D 7A 7B 7D
Chromosome

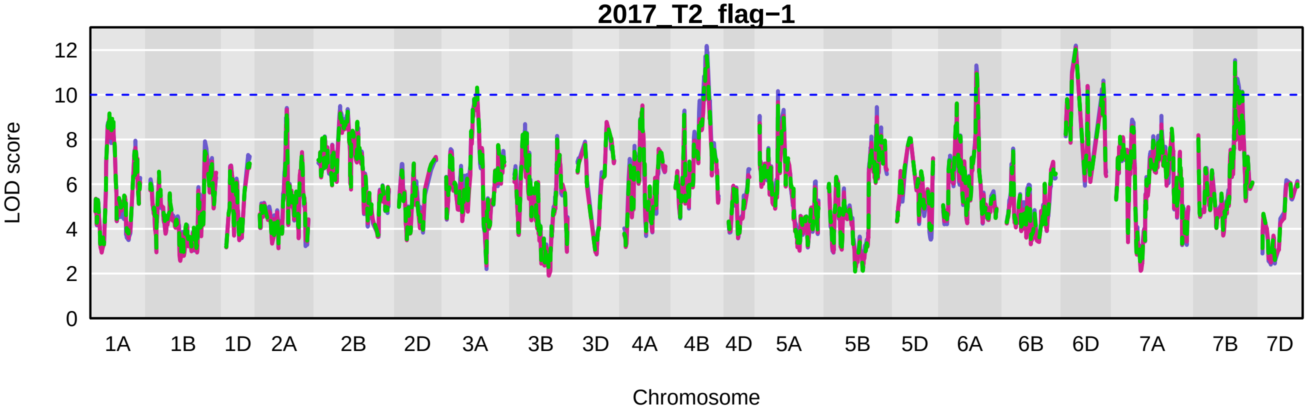
2017_T2_flag−1
LOD score
0
2
4
6
8
10
12
1A 1B 1D 2A 2B 2D 3A 3B 3D 4A 4B 4D 5A 5B 5D 6A 6B 6D 7A 7B 7D
Chromosome

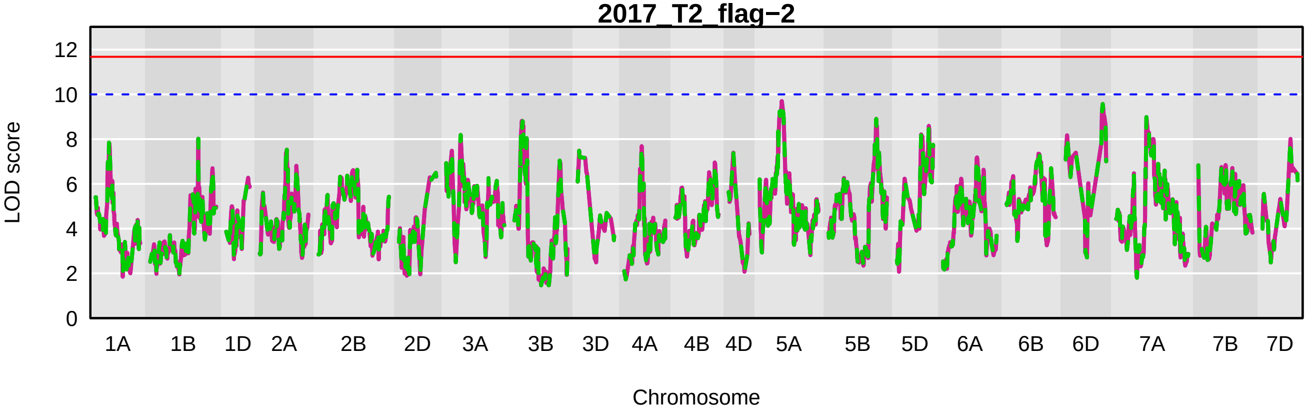
2017_T2_flag−2
LOD score
0
2
4
6
8
10
12
1A 1B 1D 2A 2B 2D 3A 3B 3D 4A 4B 4D 5A 5B 5D 6A 6B 6D 7A 7B 7D
Chromosome

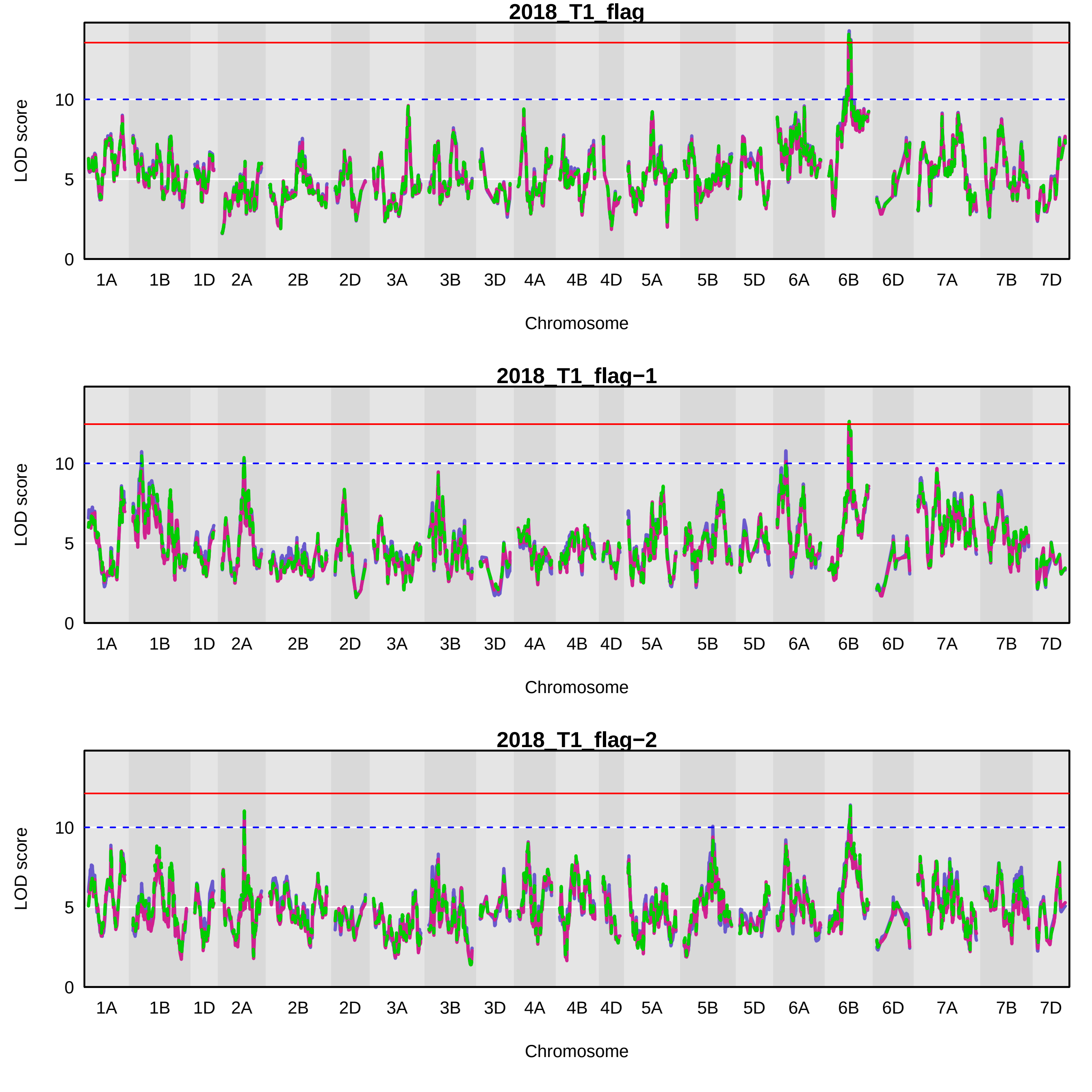
2018_T1_flag
LOD score
0
5
10
1A 1B 1D 2A 2B 2D 3A 3B 3D 4A 4B 4D 5A 5B 5D 6A 6B 6D 7A 7B 7D
Chromosome
2018_T1_flag−1
LOD score
0
5
10
1A 1B 1D 2A 2B 2D 3A 3B 3D 4A 4B 4D 5A 5B 5D 6A 6B 6D 7A 7B 7D
Chromosome
2018_T1_flag−2
LOD score
0
5
10
1A 1B 1D 2A 2B 2D 3A 3B 3D 4A 4B 4D 5A 5B 5D 6A 6B 6D 7A 7B 7D
Chromosome

Figure S3. LOD scores from QTL mapping for septoria tritici blotch (STB) resistance in the NIAB Elite MAGIC population, displayed for each dataset. Each of the individual dataset contains the LOD scores of the genome scan using the Haley-Knott regression (blue line), linear mixed model (LMM; violet line), and leave one chromosome out (LOCO; green line) model. The "red" line indicates 5% level of genome-wide significance threshold based on permutation test, while "blue dotted line" represents arbitrary threshold for all the datasets (LOD = 10.0), determining weak QTL.
