# Supplementary material for: Genetic Analysis Using a Multi-Parent Wheat Population Identifies Novel Sources of Septoria Tritici Blotch Resistance
Source: Genes (Basel). 2020 Aug 4;11(8):887. doi: 10.3390/genes11080887 (PMC7465482; doi:10.3390/genes11080887)
Supplement: Supplementary file 1 [file genes-11-00887-s001.zip › Figure S4.docx]

**
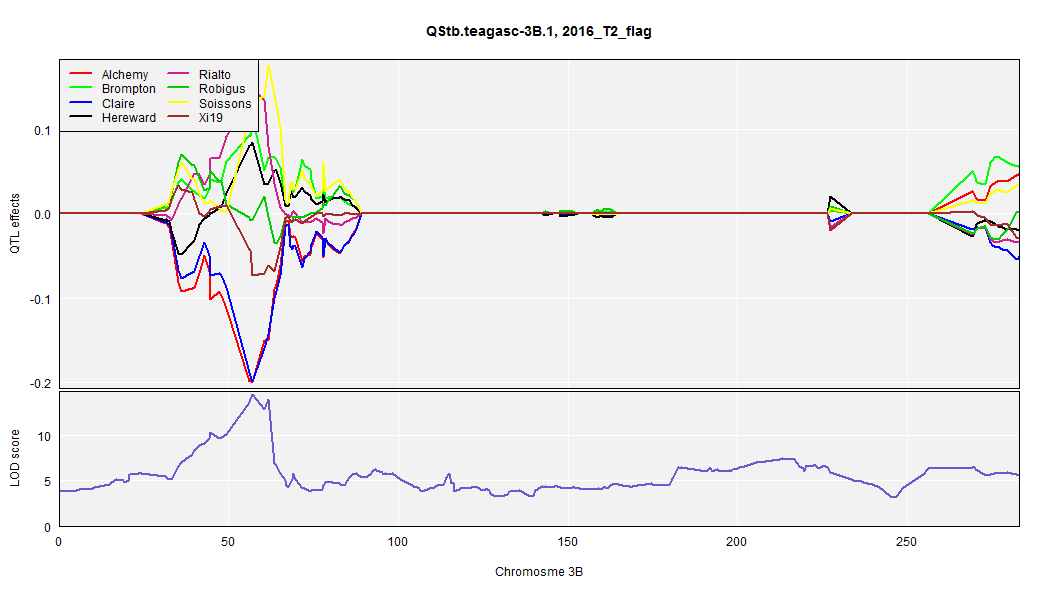
**

**
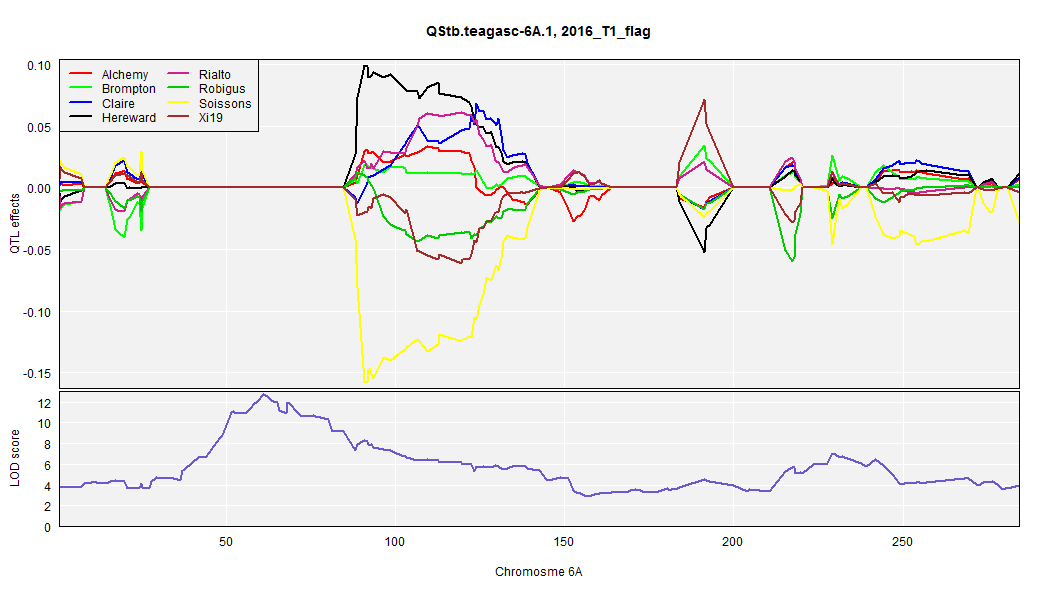
**

**
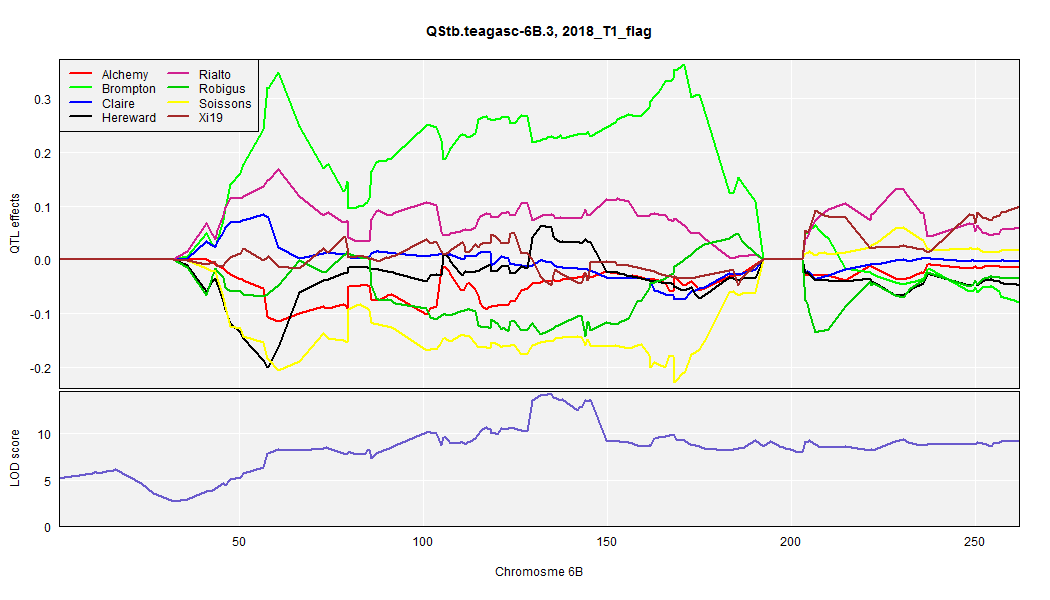
**

**
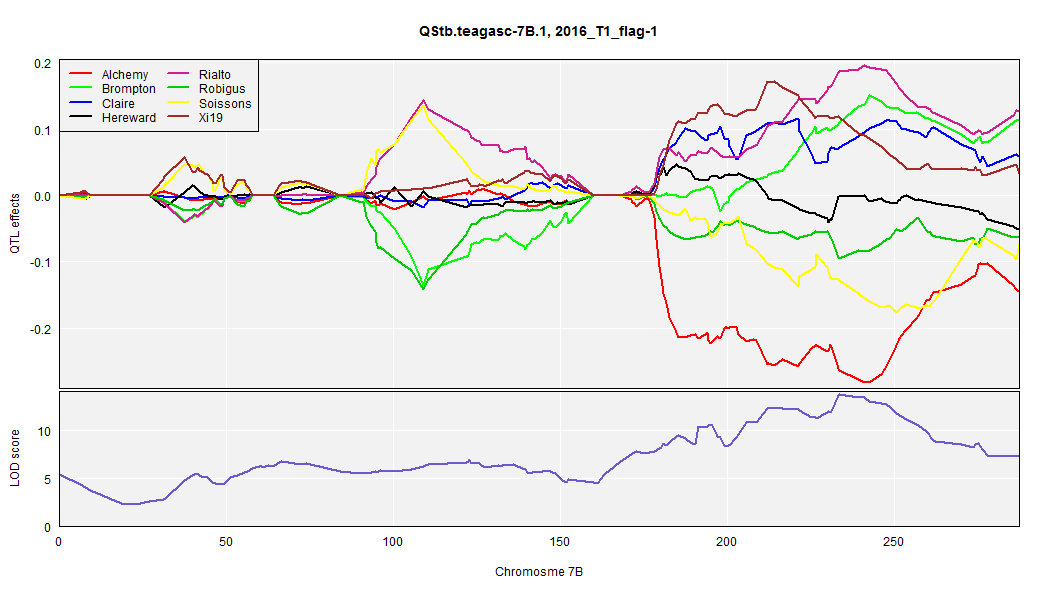
**

**Figure S4.** Estimated allele effects at different QTL for STB resistance in the NIAB Elite MAGIC population across the dataset. In each of the individual dataset, the upper panel represents BLUPs of the eight haplotype founder effects, along with the LOD curve on the respective chromosomes. The title of each figure represents QTL and dataset in which it was detected. The LOD scores of the genome scans represented here were determined by Haley-Knott regression.
